# Supplementary material for: Comparative Phylogenomic Analysis Reveals Evolutionary Genomic Changes and Novel Toxin Families in Endophytic Liberibacter Pathogens
Source: Microbiol Spectr. 2021 Sep 15;9(2):e00509-21. doi: 10.1128/Spectrum.00509-21 (PMC8557891; doi:10.1128/Spectrum.00509-21)
Supplement: SUPPLEMENTAL FILE 1 — Supplemental material. Download SPECTRUM00509-21_Supp_1_seq8.pdf, PDF file, 1.3 MB [file spectrum00509-21_supp_1_seq8.pdf]

Supplementary Materials

for

Comparative phylogenomic analysis reveals evolutionary genomic changes and novel toxin families in endophytic *Liberibacter* pathogens

Yongjun Tan[1], Cindy Wang[1], Theresa Schneider[1], Huan Li[1], Robson Francisco de Souza[2], Xueming Tang[3], Kylie D. Swisher Grimm[4], Tzung-Fu Hsieh[5,6], Xu Wang[7,8,9], Xu Li[5,6], and Dapeng Zhang[1,10,*]

[1]Department of Biology, College of Arts & Sciences, Saint Louis University, St. Louis, MO 63103

[2]Departamento de Microbiologia, Instituto de Ciências Biomédicas, Universidade de São Paulo, São Paulo 05508-900, Brazil

[3]School of Agriculture and Biology, Shanghai Jiao Tong University, Shanghai, China

[4]United States Department of Agriculture—Agricultural Research Service, Temperate Tree Fruit and Vegetable Research Unit, Prosser, WA

[5]Department of Plant and Microbial Biology, North Carolina State University, Raleigh, NC 27695

[6]Plants for Human Health Institute, North Carolina State University, Kannapolis, NC 28081

[7]Department of Pathobiology, College of Veterinary Medicine, Auburn University, Auburn, AL 36849

[8]Alabama Agricultural Experiment Station, Auburn University, Auburn, AL 36849

[9]HudsonAlpha Institute for Biotechnology, Huntsville, AL 35806

[10]Bioinformatics and Computational Biology Program, College of Arts & Sciences, Saint Louis University, St. Louis, MO 63103

Correspondence and requests for materials should be addressed to D.Z. (email: dapeng.zhang@slu.edu)

Content list

1. Supplementary Table 1. Detailed genome information of Huanglongbing-associated bacteria and other related bacteria that were used in this study (Fig.1 & Fig. 2).
2. Supplementary Table 2. Genomic positions of the identified *Liberibacter* prophage loci.
3. Supplementary Table 3. A list of orthogroups that were lost in *Liberibacter* pathogens (Appended).
4. Supplementary Table 4. A list of representative genes that were gained in the ancestor of pathogenic *Candidatus* Liberbacter species.
5. Supplementary Table 5. The KEGG pathways whose components (enzymes) are present in ancestral *L. crescens* but lost in *Liberbacter* pathogens (Appended).
6. Supplementary Figure 1. Gene ontology analysis (in the category of biological processes) of genes that were lost in pathogenic *Liberibacter* bacteria.
7. Supplementary Figure 2. The maps of KEGG pathways whose components were lost in pathogenic *Liberibacter* bacteria using the Blast2GO program.
8. Supplementary Figure 3. Multiple sequence alignment (MSA) of the PD domain.
9. Supplementary Figure 4. MSA of the Ntox52 domain.
10. Supplementary Figure 5. MSA of the beta-sandwich domain associated with the YdjM domain.
11. Supplementary Figure 6. MSA of the Exonuclease-Endonuclease-Phosphatase (EEP) family.
12. Supplementary Figure 7. Phylogenetic relationships of the *Liberibacter* EEP related sequences and other known EEP enzymes.
13. Supplementary Figure 8. MSA of the potential transporter (Peptidase_M75).
14. Supplementary Figure 9. MSA of the Phage_lysozyme family.
15. Supplementary Figure 10. MSA of a new protein family which contains four conserved Cysteines.
16. Supplementary Data 1. Detailed genomic information of the identified *Liberibacter* prophage loci (Appended).
17. Supplementary Data 2. HMM profiles of the newly-identified domains in this study.

Supplementary Table 1. Detailed genome information of Huanglongbing-associated bacteria and other related bacteria that were used in this study (Fig.1 & Fig. 2).

Species	Strain	Source	GenBank Accession Number	Length (bp)	Year	Reference
Candidatus Liberibacter asiaticus	gxpsy	Guangxi, China	NC_020549.1	1,268,237	2012	[1]
Candidatus Liberibacter asiaticus	psy62	Florida, USA	NC_012985.3	1,227,328	2010	[2]
Candidatus Liberibacter asiaticus	A4	Guangdong, China	NZ_CP010804.2	1,230,251	2019	[3]
Candidatus Liberibacter asiaticus	JXGC	Jiangxi, China	NZ_CP019958.1	1,225,162	2017	[4]
Candidatus Liberibacter asiaticus	Ishi1	Okinawa, Japan	NZ_AP014595.1	1,190,853	2014	[5]
Candidatus Liberibacter asiaticus	AHCA1	California, USA	NZ_CP029348.1	1,233,755	2018	[6]
Candidatus Liberibacter africanus	PTSAPSY	Pretoria, South Africa	NZ_CP004021.1	1,192,232	2012	[7]
Candidatus Liberibacter solanacearum	ZC1	Texas, USA	NC_014774.1	1,258,278	2010	[8]
Candidatus Liberibacter americanus	Sao Paulo	Sao Paulo, Brazil	NC_022793.1	1,195,201	2013	[9]
Candidatus Liberibacter europaeus	NZ1	Canterbury, New Zealand	PSQJ00000000.1	1,331,154	2018	[10]
Liberibacter crescens	BT0	Puerto Rico, USA	NZ_CP010522.1	1,522,119	2015	Not available
Liberibacter crescens	BT1	Puerto Rico, USA	NC_019907.1	1,504,659	2012	[11]

Supplementary Table 2. Genomic positions of the identified *Liberibacter* prophage loci.[1]

Species	Strain	Accession Number	LC1		LC2		SC		UT	
			start	stop	start	stop	start	stop	start	stop
Candidatus Liberibacter asiaticus	gxpsy	NC_020549.1			1118314	1137878	1223784<br>1186049	1264363<br>1223266	1173699	1185042
	psy62	NC_012985.3			1132029	1149043	1196268	9590	1184862	1195729
	A4	NZ_CP010804.2			1129881	1146896	1194901	3341	1182715	1194055
	JXGC	NZ_CP019958.1			1129886	1149448	1196849	3320	1185268	1196611
	Ishi1	NZ_AP014595.1			1127556	1144572			1180544	1788
	AHCA1	NZ_CP029348.1			1129943	1149509	1200706	3333	1185334	1196215
Candidatus Liberibacter afticanus	PTSA-PSY	NZ_CP004021.1			26536	48147	449353	488428		
Candidatus Liberibacter solanacearum	ZC1	NC_014774.1			814348	832703	176477<br>1214438	216722<br>1257724		
Candidatus Liberibacter americanus	Sao Paulo	NC_022793.1			555495	576335	749038<br>788985	787835<br>806744		
Candidatus Liberibacter europaeus	NZ1	PSQJ01000004.1			44516	62901				
		PSQJ01000015.1					1	16745		
		PSQJ01000003.1					1	13653		
		PSQJ01000006.1					173	39658		
		PSQJ01000005.1					20427	41563		
		PSQJ01000013.1					195	3703		
Liberibacter crescens	BT0	NZ_CP010522.1	588495	601703	898955	916810				
	BT1	NC_019907.1	544655	557864	850693	868545				

[1] The shading blocks indicate that bacteria do not harbor the corresponding prophage loci.

Supplementary Table 4. A list of representative genes that were gained in the ancestor of pathogenic *Candidatus* Liberibacter species.

Ortholog Groups Representative Accession	Length	Definition	Domain Architecture (Pfam + novel domains)	Function Annotation
		Toxins/effectors		
WP_012778667.1	311	hypothetical protein	PD+Ntox52	Ntox52 is distantly homologous to the EspG/VirA effectors
WP_012778668.1	459	hypothetical protein	PD+Ntox52	
WP_015452837.1	304	hypothetical protein	SP+EEP	EEP family[12] (see Supplementary Figure 6)
WP_015452856.1	163	metal-dependent hydrolase	YdjM	Homologous to alpha-toxin and the Het-C toxin domain[13]
		Transporters		
WP_012778500.1	201	SCO family protein	SP+SCO1-SenC	periplasmic copper binding protein [14]
WP_012778517.1	118	EamA family transporter	SP+EamA	small multidrug resistance protein
WP_012778542.1	491	NTP/NDP exchange transporter	SP+TLC	NTP/NDP transporter
WP_015452442.1	260	metal ABC transporter permease	ABC-3	Ion ABC transporter
WP_015452443.1	240	metal ABC transporter ATP-binding protein	ABC_tran	Metal ABC transporter
WP_015452444.1	294	zinc ABC transporter solute-binding protein	SP+ZnuA	Zinc ABC transporter
WP_102134465.1	418	peptidase	SP+Peptidase_M75 (wrong annotation)	Iron transporter[15] (see Supplementary Figure 8)
WP_015452664.1	314	cation transporter	Cation_efflux	Cation transporter
WP_015452813.1	202	LysE family translocator	LysE	Lysine translocator
WP_015452909.1	242	outer membrane beta-barrel protein	SP+beta-barral	A beta barrel membrane protein
WP_015452959.1	225	hypothetical protein	SP+ beta-barral	
WP_015452999.1	420	dicarboxylate/amino acid:cation symporter	SDF	Sodium:dicarboxylate transporter
WP_015452595.1	195	ABC transporter substrate-binding protein	SP+SBP_bac_6	ABC transporter
WP_015824913.1	107	hypothetical protein	SBP_bac_8	periplasmic iron-binding protein
		Enzymes for DNA/RNA synthesis and regulation		
WP_109298658.1	1781	DUF4011 domain-containing protein	DUF4011+AAA_11 +RNA-Helicase_SFI +REase_AbiJ-DUF2726	Replication/transcription
WP_015452495.1	445	RsmB/NOP family class I SAM-dependent RNA methyltransferase	NusB+Methyltr_RsmB-F	16S rRNA (cytosine(967)-C(5))-methyltransferase [16]
WP_015452499.1	471	exodeoxyribonuclease I	35exo_RnaseT.1 +Exonuc_X-T_C	exodeoxyribonuclease I involved in DNA recombination and repair [17]
WP_015452977.1	83	helix-turn-helix transcriptional regulator	HTH	transcriptional factor
WP_015452998.1	483	deoxyribodipyrimidine photo-lyase	DNA_photolyase +FAD_binding_7	DNA photolyase [18]
		Enzymes for small molecule metabolism		
WP_015452887.1	171	lysozyme	Phage_lysozyme	glycoside hydrolase[19] (see Supplementary Figure 9)
WP_015452479.1	250	5'/3'-nucleotidase SurE	SurE	Survival protein; metal-dependent phosphatase [20]
WP_015452406.1	216	MBL fold metallo-hydrolase	Mbetalac	beta-lactamase; antibiotic resistance [21]
WP_015452826.1	152	acyl-CoA thioesterase	4HBT	thioesterase [22]
WP_015452942.1	298	polyprenyl synthetase family protein	polyprenyl_synt	polyprenyl synthetases
		Transmembrane proteins		
WP_012778543.1	61	hypothetical protein	TM	Transmembrane protein
WP_015452822.1	151	hypothetical protein	TM+TM	Transmembrane protein
WP_015452847.1	302	DUF4065 domain-containing protein	DUF4065+TM+TM	Transmembrane protein
WP_047263992.1	119	hypothetical protein	TM+Unknwon region	Transmembrane protein
		Function unknown proteins		
WP_015452797.1	229	hypothetical protein	SP+4Cys	May be a novel toxin (see Supplementary Figure 10)
WP_012778451.1	98	hypothetical protein	DUF167	
WP_047263804.1	189	hypothetical protein	DUF6456	

Supplementary Figure 1. Gene Ontology analysis (in the category of biological processes) of genes that were lost in pathogenic *Liberibacter* bacteria using the Blast2GO program.

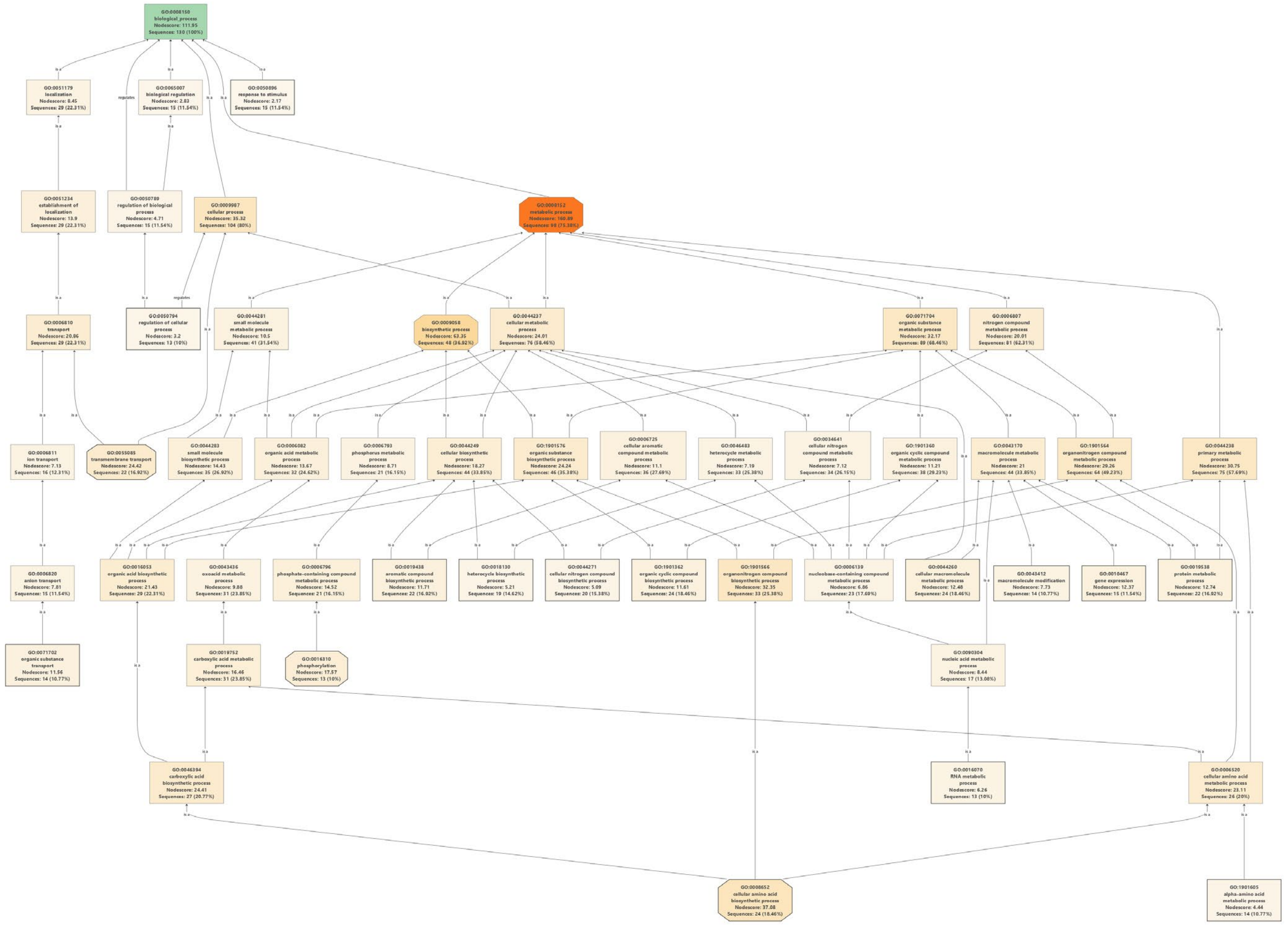

Supplementary Figure 2. The maps of KEGG pathways whose components were lost in pathogenic *Liberibacter* bacteria.

a. KEGG pathway of Phenylalanine, Tyrosine and Tryptophan biosynthesis.

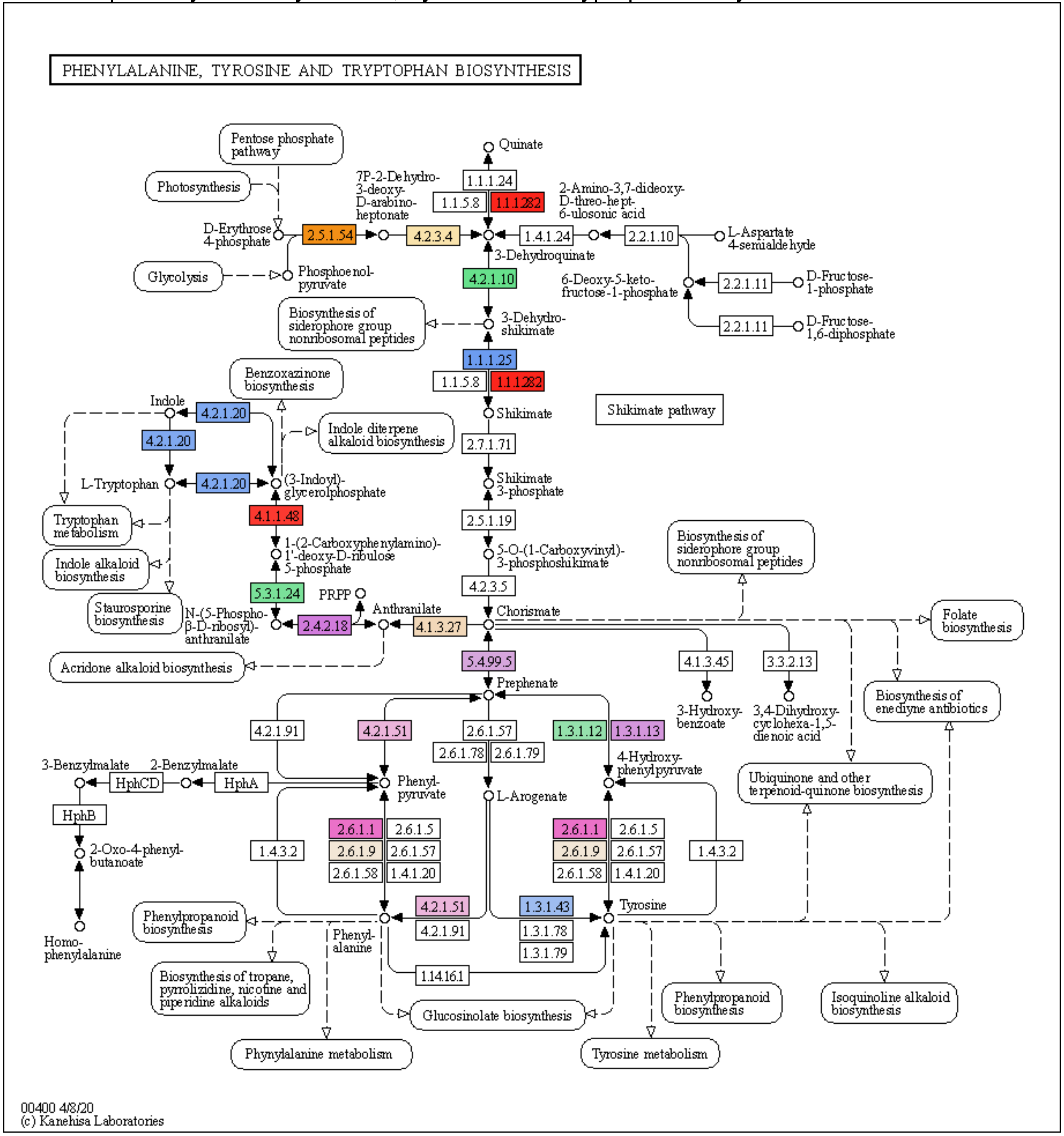

b. KEGG pathway of Histidine metabolism.

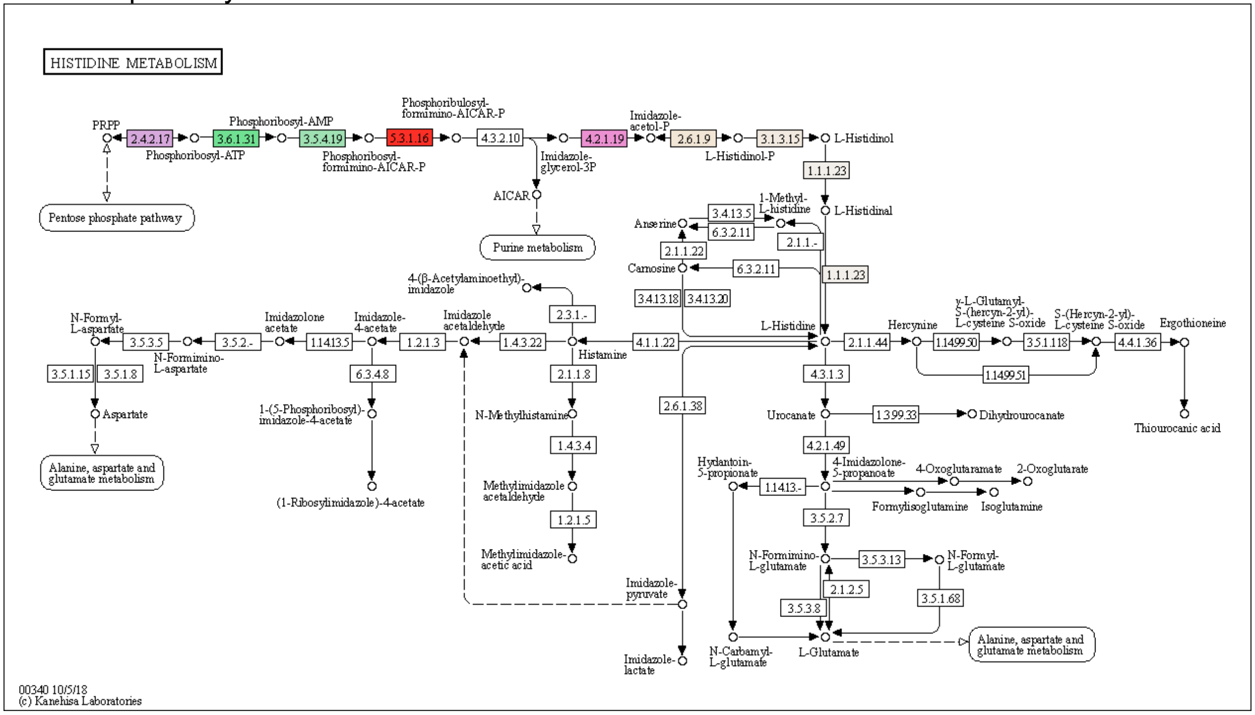

c. KEGG pathway of Pantothenate and CoA biosynthesis.

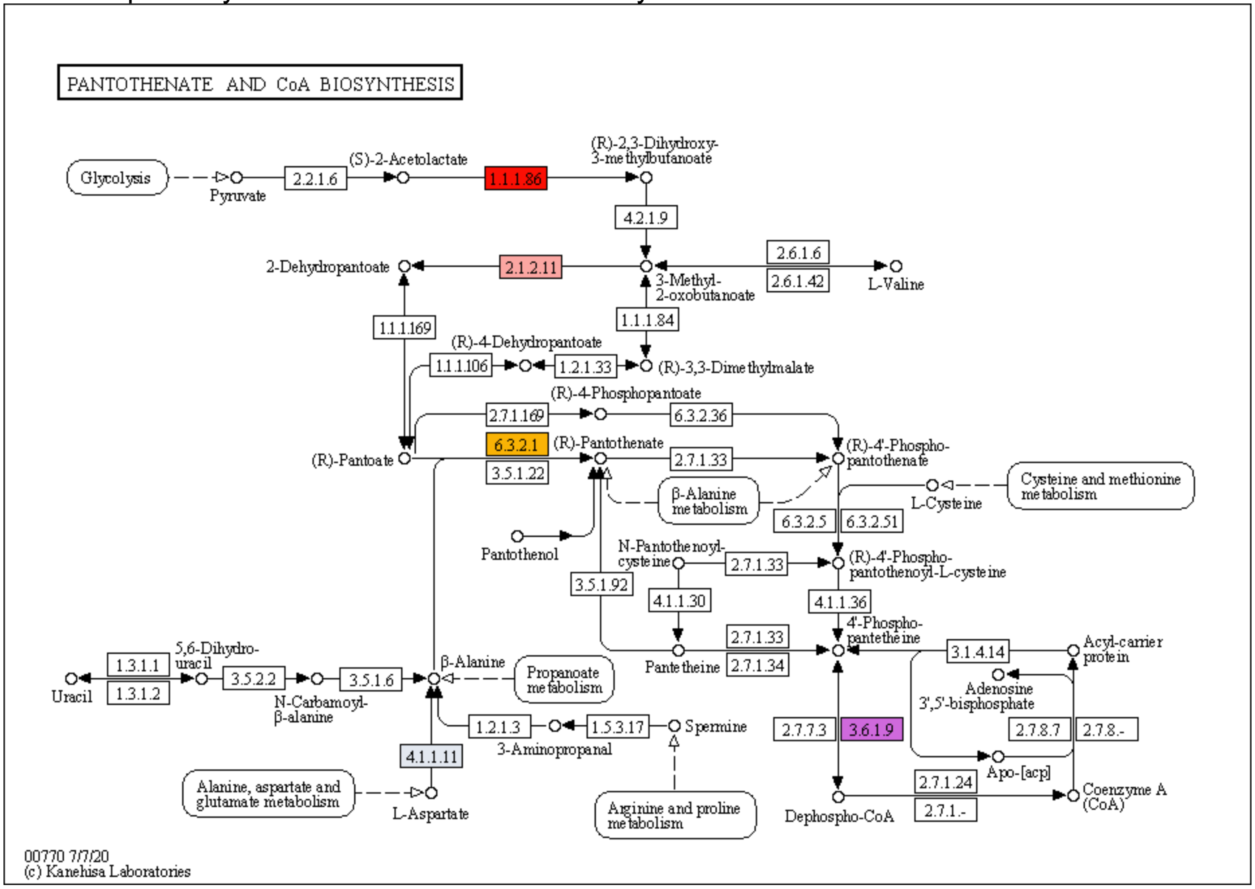

d. KEGG pathway of Purine metabolism.

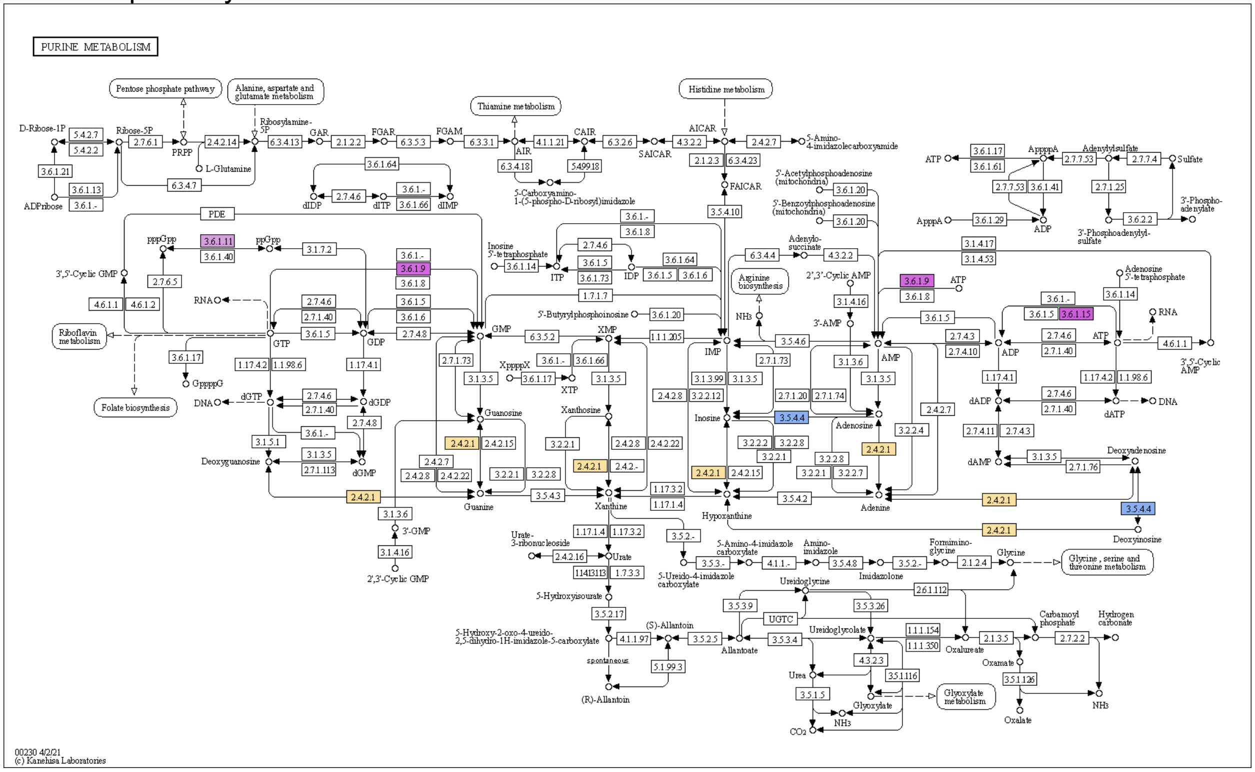

e. KEGG pathway of Novobiocin biosynthesis.

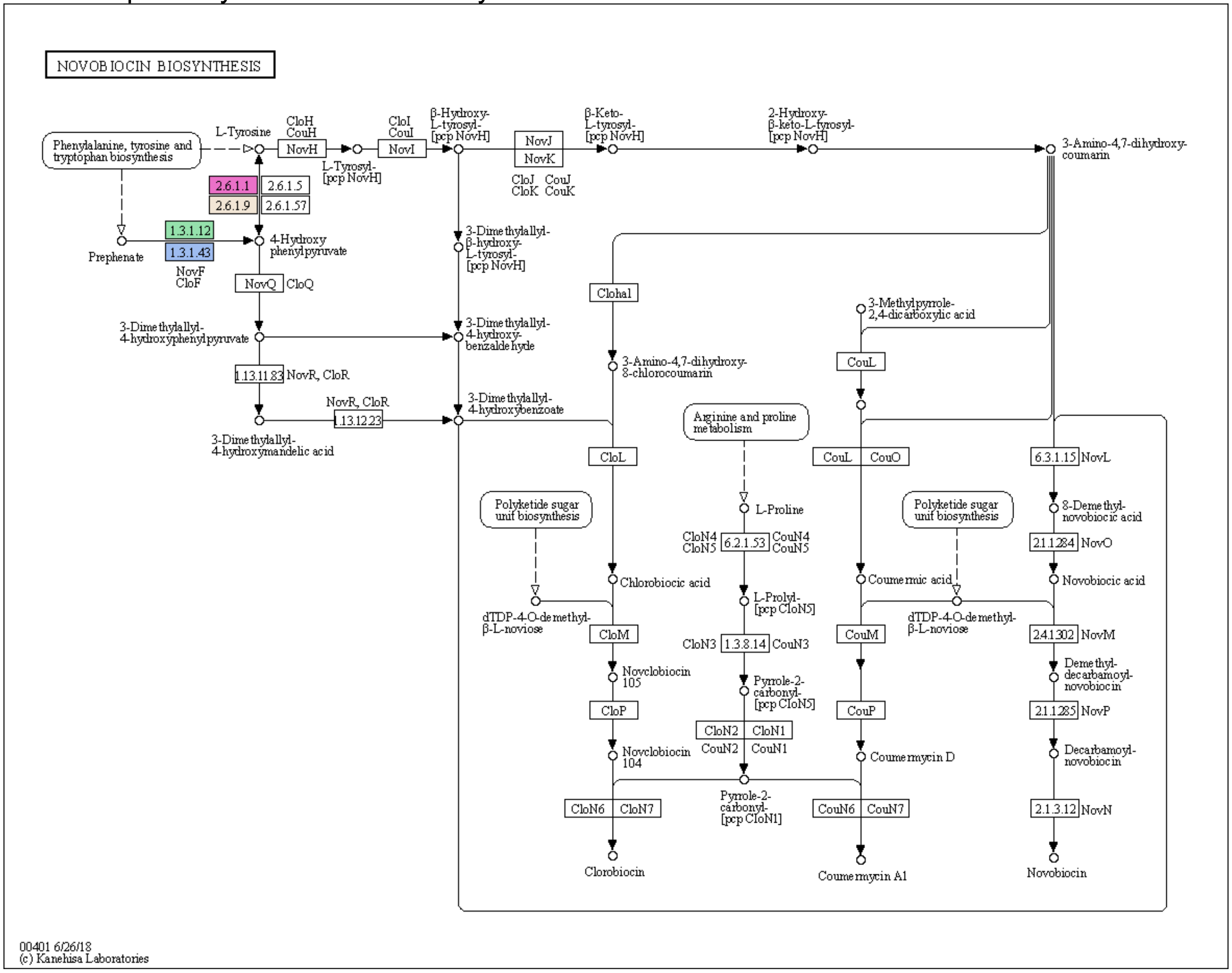

f. KEGG pathway of Thiamine metabolism.

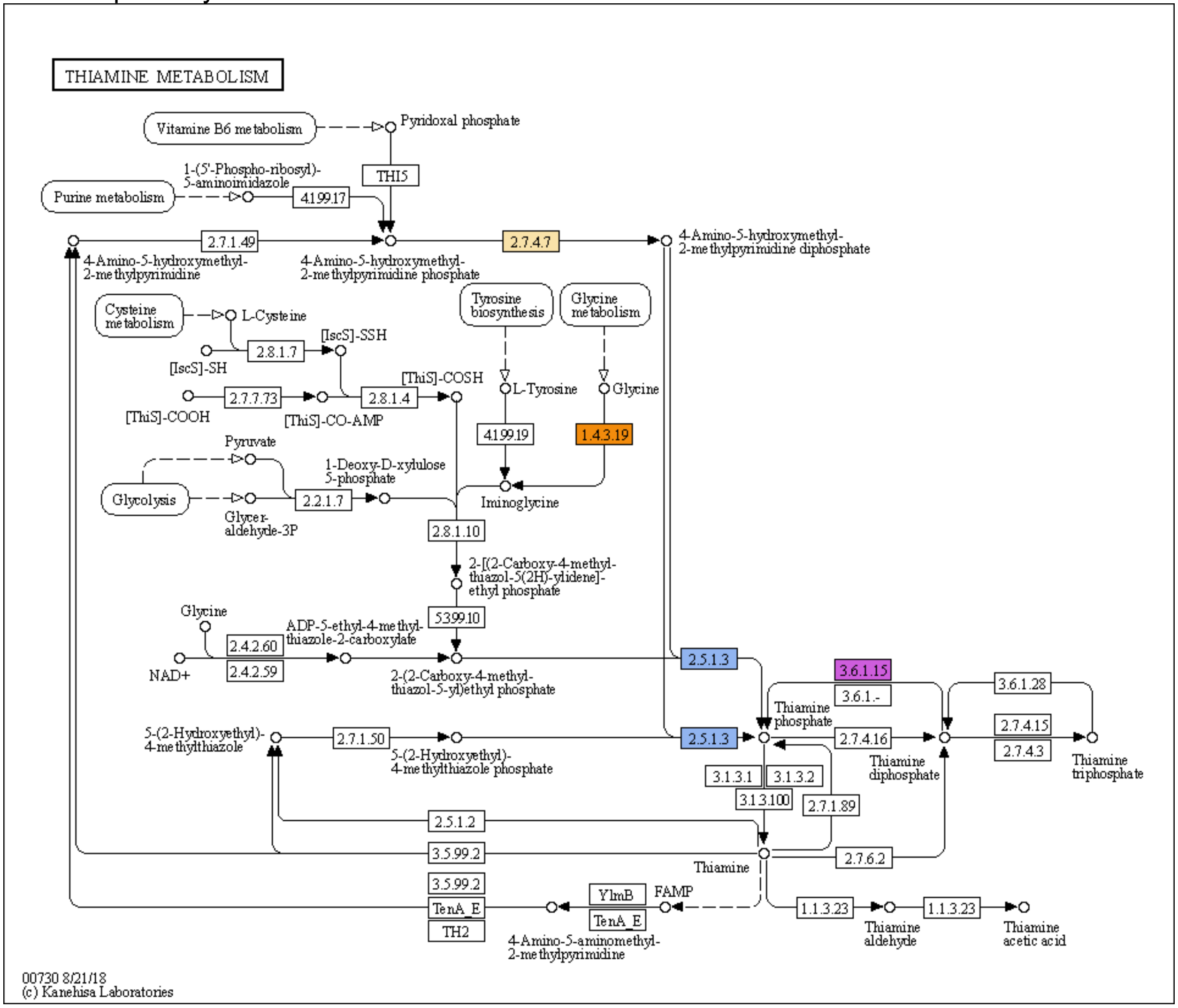

g. KEGG pathway of Cysteine and Methionine metabolism.

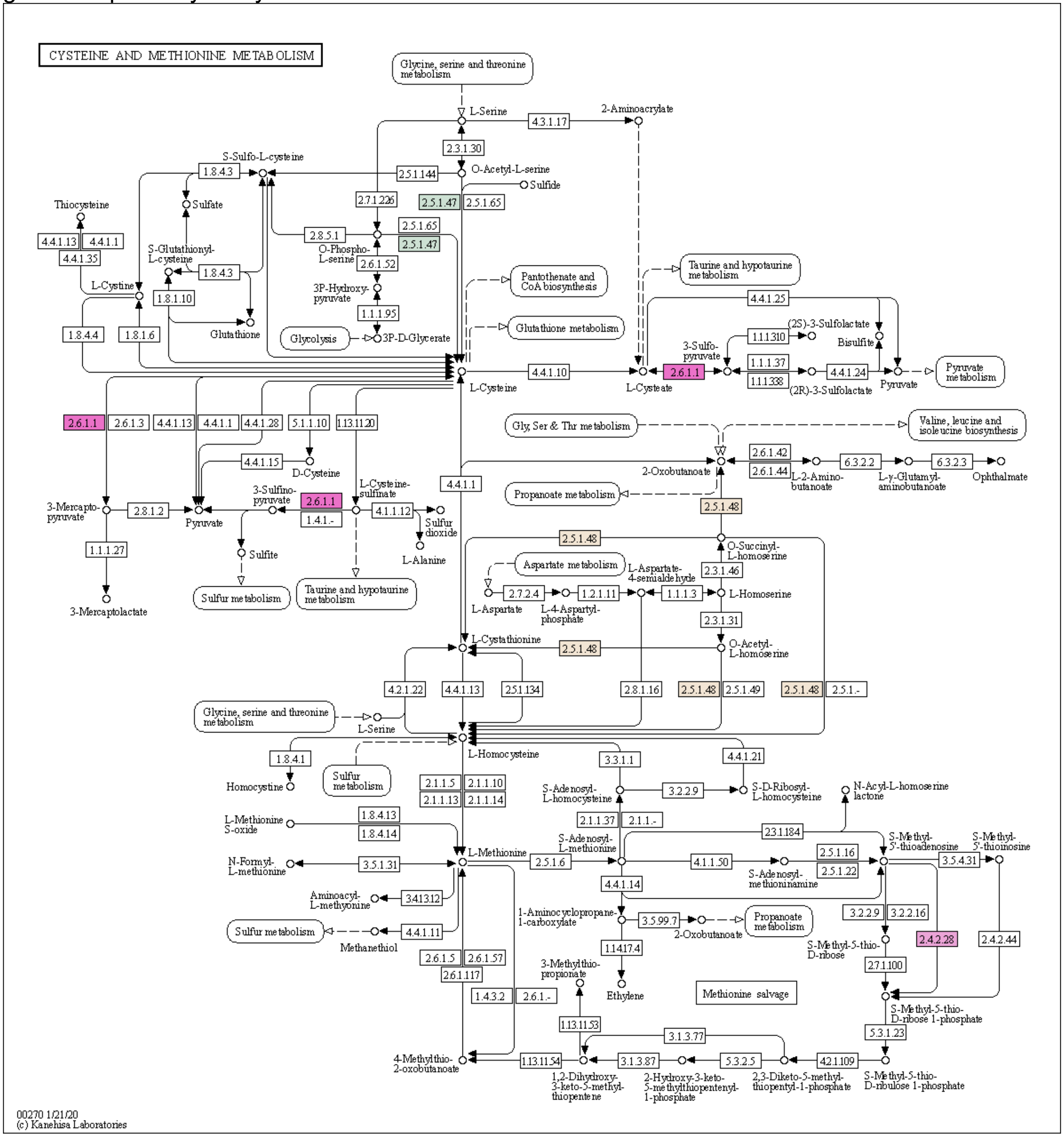

h. KEGG pathway of Arginine and Proline metabolism.

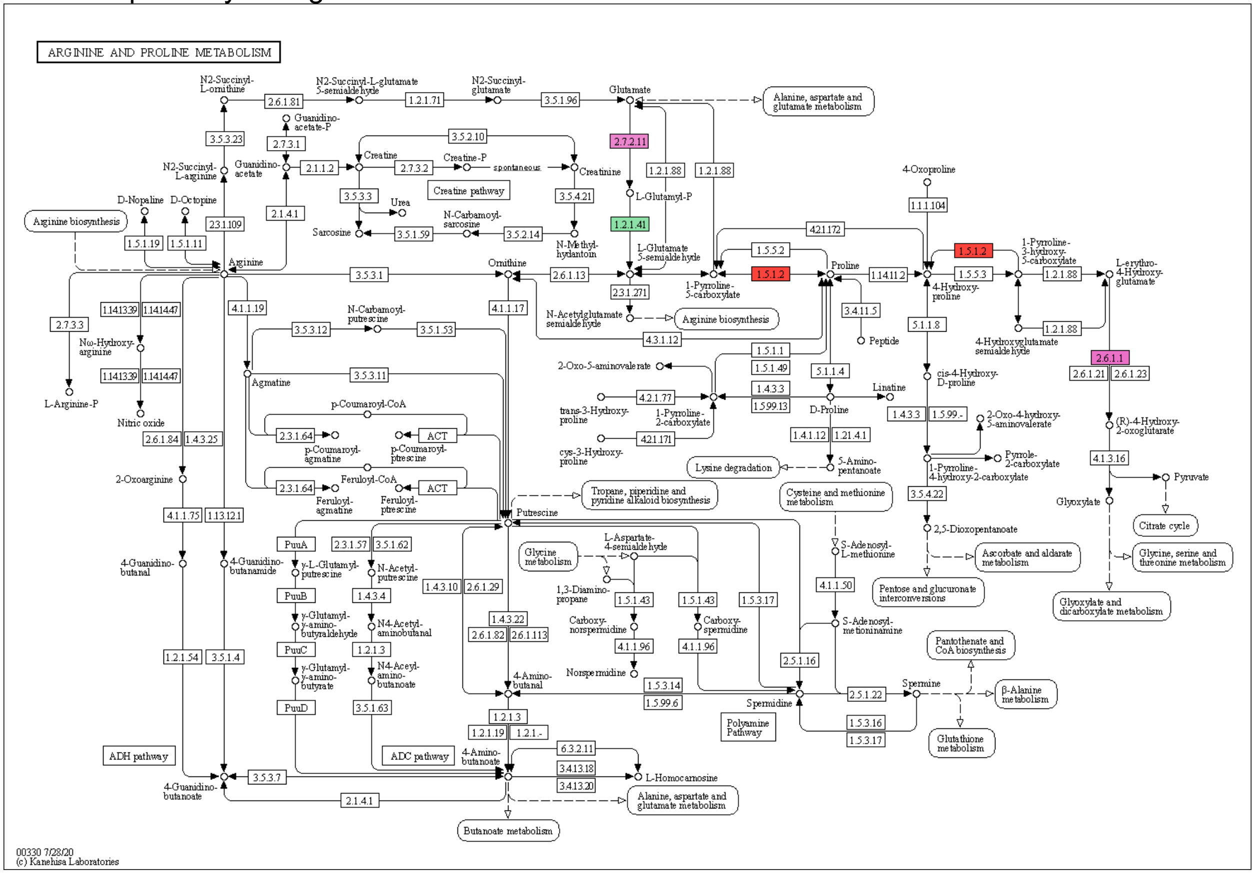

i. KEGG pathway of Nicotinate and Nicotinamide metabolism.

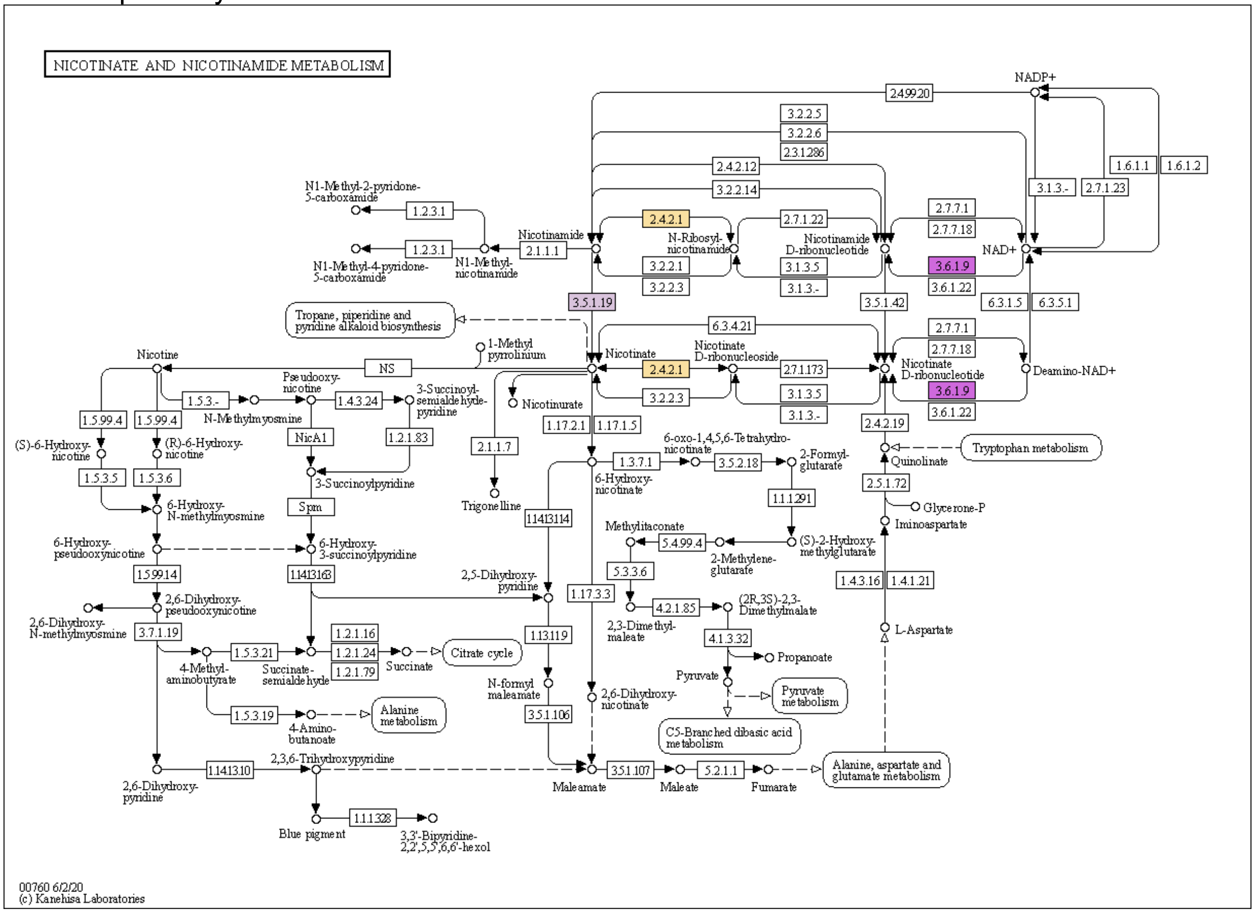

j. KEGG pathway of Tyrosine metabolism.

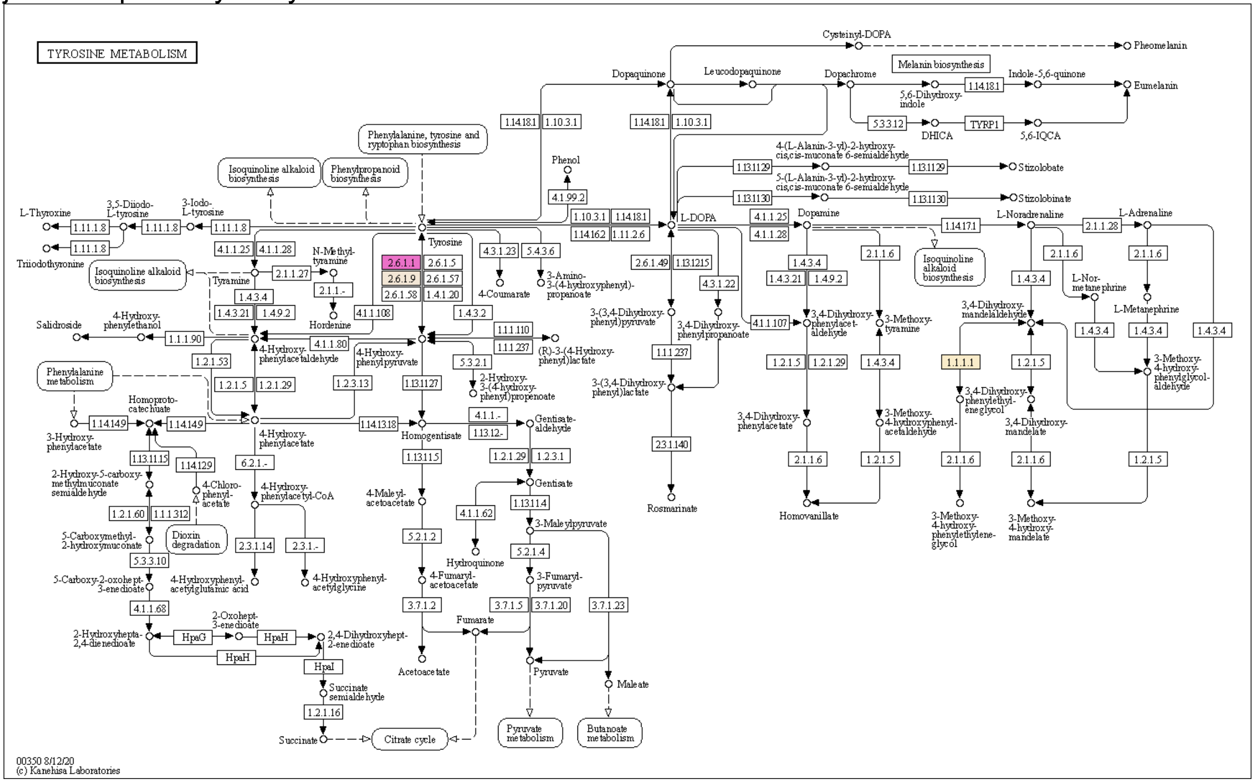

k. KEGG pathway of Glycerophospholipid metabolism.

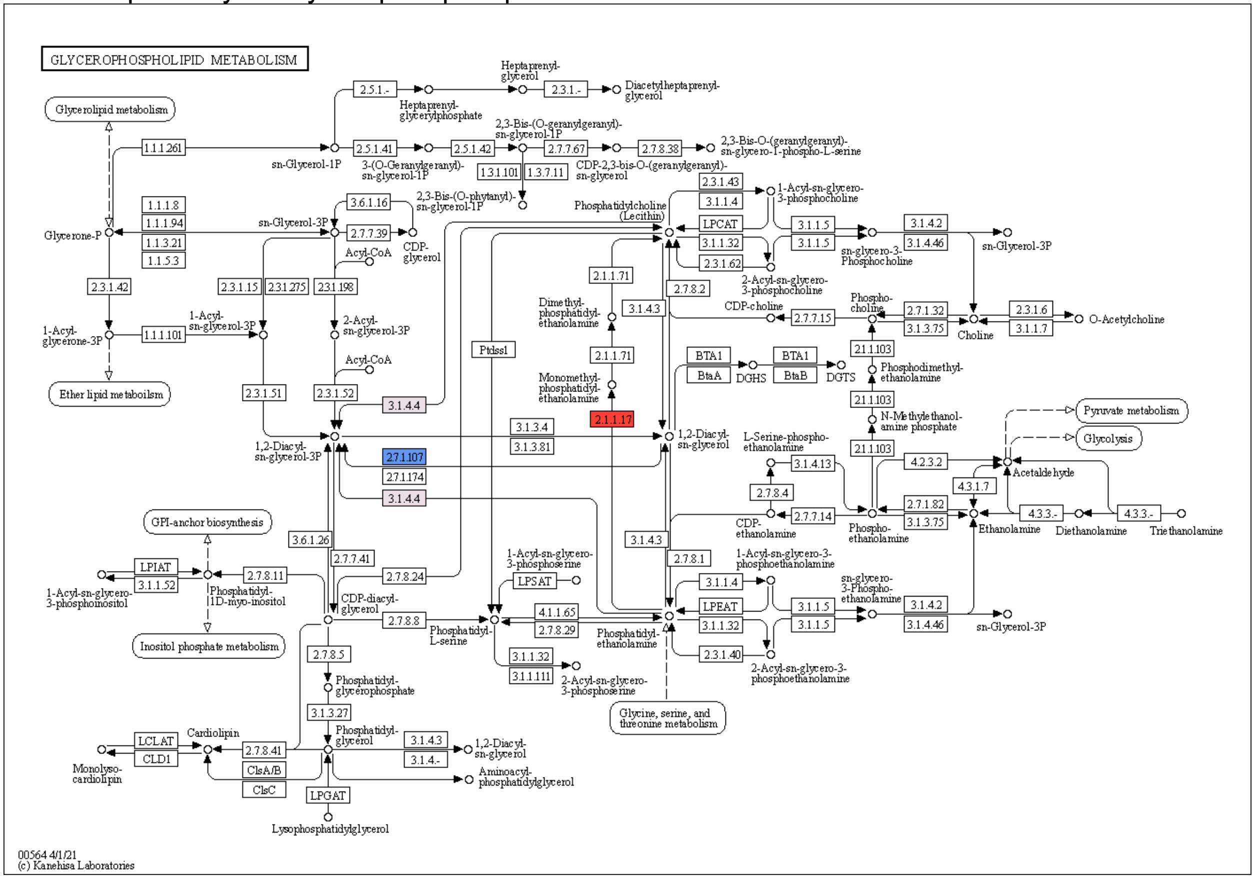

I. KEGG pathway of Fatty acid biosynthesis.

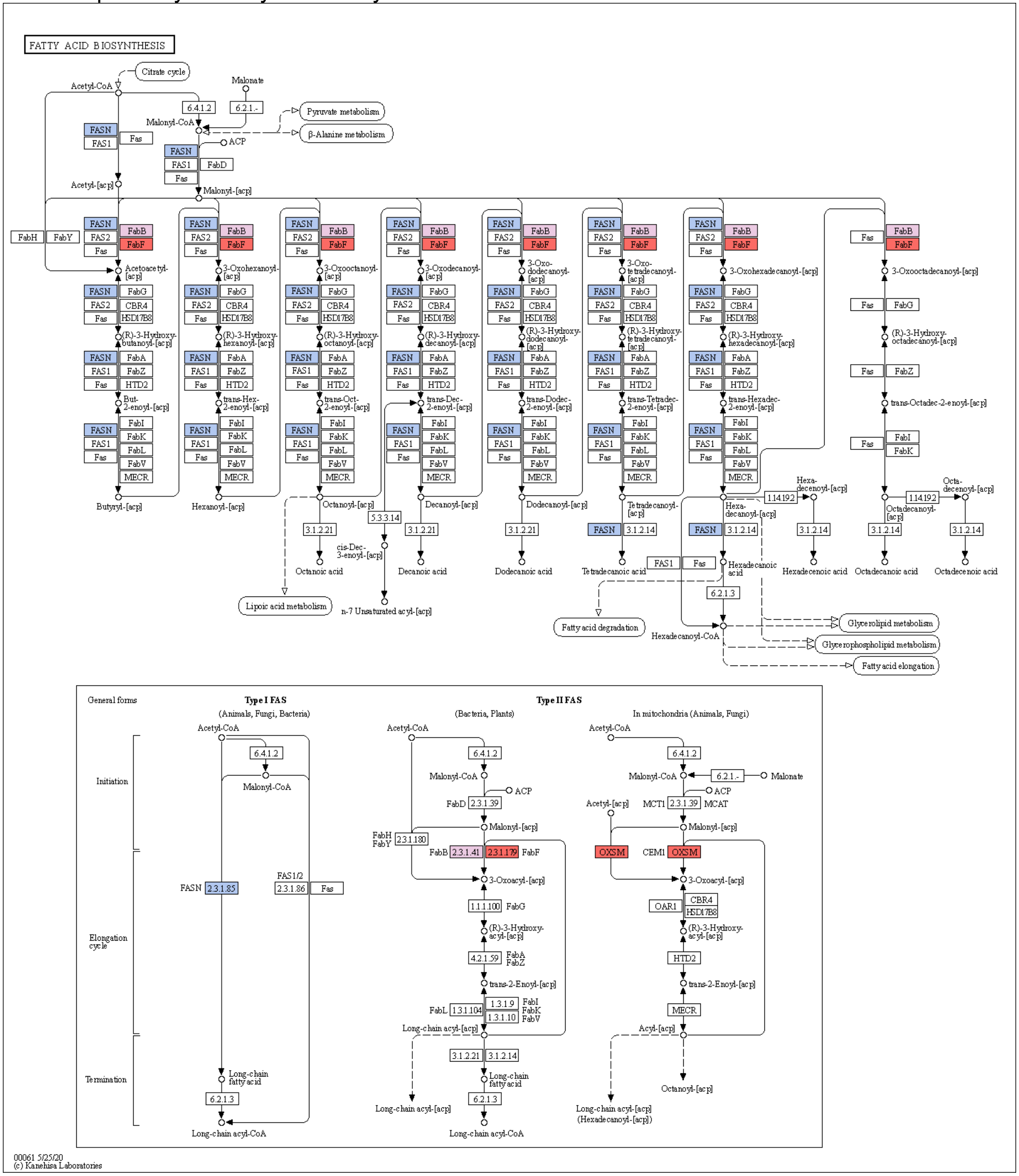

Supplementary Figure 3. Multiple sequence alignment (MSA) of the PD domain. The sequences in MSA are labelled by their species abbreviation followed by NCBI accession number. The secondary structure predicted by JPRED is shown above the MSA, while the consensus result calculated by a custom perl script is displayed below the MSA. The MSA was colored according to the consensus in 80% of the sequences based on 7 amino acid classes (see Methods section for details). Coloring theme: the amino acid involved in either catalytic activities or specific function is highlighted in red background with white font color and bold font style; any amino acid present above the threshold is highlighted in black background with white font color and bold font style; hydrophobic (h) in yellow background with bold font style; aromatic groups (a) in orange background with bold font style; big amino acids (b) in light grey background with bold font style; small amino acids (s) in green background with bold font style; polar (p) group in blue background with bold font style; charged (c) amino acids in pink background with bold font style. The sequence, CLaf_WP_148407341.1, consist of two protein sequences, WP_052775023.1 and WP_148407341.1, which originated from the same gene in *C.* Liberibacter africanus.

```
Secondary Structure   HHHHHHHHHHHHHHH   HHHHHHHH         HH HHHHHHHHHHHH        HHH   HHHHH       HHHHHHHHHHHHHH
CLas_WP_012778668.1   STKVKDVAVDVAVSM-IPIYGTYREFKKGNYG---WG-IVGAISDAALLI-PVVGYGARAA---INLVRGGSI---ALKAGTAGTMIAAKEACT
CLas_WP_012778667.1   --------------M-IPVYGTIQEFKKGNYG---WG-ALGIVSDVALLA-IPAAYLGKVL---FGLVRGSSI---ATKIATTGIATVVQEATV
CLaf_WP_148407341.1   LEKAKECTKEAAISM-IPVYGTIQEFK/-------MG-ANALPSETKTLL--PSKNSAKIS-----VAKS------ESKLDNIYLSGKKTINK-
CLso_WP_013461761.1   ISTLESIAKKSLIAA-IPIYGTIQAFKEKESG---WG-ILGITTDVLTLI--GIGYGIKGA---AALIRGSS----AAATAAMAAGTSTAIEGG
CLso_WP_044054101.1   KNEIYHLAKEVAISS-IPIYGTIQAFKKGEIG---WG-IFGIVTDALLIV-PAIGVGAKLA---VNFI--------ARRSKIASTTMRSTATIA
CLso_WP_013461760.1   SSKIGETAKEALLSL-IPIYGTIQSFKKGEIG---WG-IFGAITDVLTLV-PVVGYGAKMV---GALARGGNA---AIKISKAGAIAASATAST
CLam_WP_007557209.1   SSDFFSNAKDVMVNL-IPIYGTINEFKKGNTG---WG-IVGVITDALILV-PVFGYGIKVV---ATLARGGAKV--AENAIKAKTIINDFKVLQ
CLam_WP_023466137.1   MDKVSQVAKDVLMSS-IPIYGTIQSFKKGEIG---WG-IFGIATDILTLV-PVLGSSAKLA---FAASIATRE---AKGLLATAKAVKATSDVV
CLam_WP_007556842.1   IKNILEIAKNAAINS-IPIYGTIQAFKKGETK---WG-IFGIVTDVLTLV-PIIGIGSKLI---GSVIKAGSI---TTKFGRTMKGGKASIELK
CLam_WP_023466135.1   IDNILNIAKEAAISS-IPIYGTIQSFKKGEIG---WG-IFGIATDFLTLV-PALGFSVKFA---GSLIRGGGV---AMKVETKVVAETMKIANV
CLeu_PTL86945.1       SSNIFYIAKEVFTSM-IPIYGSIQAFKKGNTL---YG-LFGIIADILTLI-HIMALGDKLE---GFLIQG------CKAIVSSIVLV-------
CLeu_PTL86946.1       MSTFFHVAKEVGLSS-IPIYGTIRSFKNGEIG---WG-IFGIVTDVLTVI-PVLGVSAKLA---GSLIRGGGA---AIKAGATTAEVIAKTGAA
CLeu_PTL86771.1       ANQITHAVKEALIST-IPIYGTIQEFKKGEIR---WG-IFGAVTDVLLLV-PVLGCSAKLI---GSLARGAKVAKNAIEIGKTGYVASKVIHGG
Lsp._MBA5723779.1     ESPYLEVAKEVGISM-IPIYGTIHEFQKGNIG---WG-IFSAVTDALSLI-PVIGAGCKAT---GALIRGGET---AIKGIEACVVTTESINTT
Lsp._MBA5723780.1     ESPYLEAAKEIGISM-IPIYGTIHEFQKGNIG---WG-IFSAVTDALSLI-PVIGAGCKAT---GALIRGGET---AIKGIEACVVTTESINAT
Ssym_NIH12428.1       QVSAGEISKEVALSM-IPIYGTVREFQKGNIG---WG-IFGAITDALTLI-PVIGAGAKAI---GTAIRGGSAAVKALRVGLTTAEVTSKITT-
Xalb_CBA15982.1       AKTTYEKVTDFALSL-IPFYECGKNIYQGKIE---EA-VIPCTVDVLGLI-PLAVPAGRVV------MKFGE----ALSFSALRALRQSSA---
Vcam_WP_103412121.1   VTTDRDILFMVLQAF-IPVWGTVESFQRGDKM---GS-VLGLTGDFMFFV--GVGTGIKSA(4)QLLLKRS-----KHLFGRGVSLAVKGI---
Vaqu_WP_126575537.1   REKARRFSKSIGKAI-LPLWGTIDDFKMGNNS---AG-VAGVVGDIMTFV-PIFKASASAA-----VGRDVTL---SLTLKALKNSSKSFARGI
Srub_WP_163833448.1   QQTARQQSMEMLLDMLVPFYGFAQAIKKGNVG---EA-IISGFADFLSLI-PVAGAAGKLG---GTALKTGF----KVANIAARQALKAGLKTG
Pwei_KMN12671.1       EHPVTRQILNLAVNS-IPLVSSIKSFAKGDIA---GG-VTDLTLDIFGFIVPGVKA-GKAAG---GLAKGAST---TSRYITLASKVAKALIVT
Pvir_WP_029242134.1   WAKEREYADTAARFL-IPFYGCIQDLRAGEHS---AGVIVGCVLDAAFALVPLGQFAGATA---RIVMRAGEL---SVLSITRLTGKALGR---
Psp._WP_158835469.1   IDGEADKWIGSALAF-IPFYTAIKNFIAGNTA---MG-LFYASLDVFGLM--LGGTGNRLV---SELVRSAAG---KGTLKSLAALAQAPS---
Psp._WP_116628907.1   KLKTDKSLKDTLIRM-IPLVAAAQDAKAGNYG---EA-AVDLAFDAFGFL-APELKLIEAGG--SALAKLG-----SREVESLASSGAKGEV--
Prho_MBB5004468.1     SREARTRAHNFLLGF-VPFVGAYQEFKAGNIG---SG-LANLAVDTVGVA-IGAAGPAR------GLIRSAK----ALAFKPLKGIARRLTATV
Pkor_WP_077573129.1   ERTKGEKIATYFVDLAVPFKKCIEDISSGEHNRVVDG-IYGCLMDAIALG-GAFYGAGV---------KALSI---SAKTISAASKAARVTKLV
Pfre_KAF2390978.1     IKETFDSRFDTVKSF-IPFWGSIEDLTSGDTDRMFGG-FLGLMMDVVSFALPIGKFVSGSV----RLVKLASAASVAGQLSTKATLPSFSTLT-
Pflu_WP_044273868.1   EKEKQEFFRNLVLGL-IPGGSALWNLAHGNYK---DA-AADIIFDIVLYA--TTAGLGKAGS---GAARAG-----SSRFGKYLTAGKSTAGVG
Pcos_SEE43809.1       EVTVFEQIQAVTRAL-IPFASAIDSFQKGKIG---QG-LIFLAFDIFGFVVGGVGALNRIS----KVVKVGGSVGGKAGRLARGLLSAANPFAG
Psil_RKP54526.1       IQKRNAALVDFLKSL-VPFHDFIQHVRAGQSS---EA-VGALGRDVIALI-PLARAIGKGVTGLTRLARIGGA---TARAGSANAAGAIT----
CRin_EFL91365.1       EATIIEKIADFLKHL-IPFYDAIKNLQDGKKL---EA-AGYFVLDLLMFL-PLIGEVGKLS--------VGM----SEVLISAANISR------
Balb_TKI06771.1       HKHVLHAARDFLLSL-LPFYSCVDGVKTDPLG---ET-LFDCGMDIVSLL-PVLGPLSKMT(4)TVFIRGLQK---TGREALMMMAARKSLGNI
Bfla_ANN79506.1       GQTISEKLAEAERGL-LPFYATYRAIRDGDVR---GA-ILSGLMDVATFL-PAIGAGLKLGMTASAAIKA------GLEAGLQG----------
Consensus/80%         .pp..p.h.phhbsh.bPbassbp.bp.Gph.....s.hhshhhDhhhbh..hhhh.schh.....hhcss.....s..h..h...........
```

HHH → alpha helix

EEE → beta strand

Supplementary Figure 4. Multiple sequence alignment (MSA) of the Ntox52 domain. For coloring themes refer to the Supplementary Figure 3 legend.

```
Secondary Structure  HHHHHHHH        HHHHHHHHHHH  EEEE  EE       HHHHHHHHHHHH     HHHHHHHHHHHHHHHHHHHHHHHHHH          EEE
CLas_WP_012778668.1  LFRDFQGLCG----KNIDNQFILDLNR-ASFIFNGKKLARD---NSAEAIQKLMNQFAK-NPKQLQLISSYANQSIF-ADSVVHLMQSIPEFAK-YASKS
CLas_WP_012778667.1  YFRDFKLLG-----TNKVYKNLLDASRATEFIIDGKKINID---SAQNMLAELNKIFPK-DFEKVQLISSYAHEHIF-CKPFTKDLLNL--ANKNIYQLS
CLaf_WP_148407341.1  LFKSFKSLPA----KNLDHQFLIDLPR-SSYIINGKELARD---NSSKAVKKLISLFPN-SYQKLQLISSYATQSIF-SESIIHLTASIPEFKF--HGSS
CLso_WP_013461761.1  YFYDFKLLS-----PNKAYKGLRDASKASEFIVNGKKINID---TPEKMLENLKEIFPN-DFEKVQLISCYAHEGIF-DAPFTHKLFSINNPKN--YTGV
CLso_WP_013461760.1  LFRNFKQLNK----NELDQQFLLDLNR-AEYIINGKNMRDT---NQKSQLAYLQKTFAN-DPQKLQIISAYAHQGIF-ADGISYLMETIPNMLS--YGSK
CLam_WP_023466137.1  MLEDFHKLG-----DSINNQLSLDLTR-STYIINGKVINKS---NPEKMIEEIKDMVHQ-DNRRLQLISTFSNQAIF-PNLSIHIMKTDPRLGQQLYGSK
CLam_WP_007556842.1  MFKDFKDLGN----LQLSDKFIRDAAR-SKYEINGKIGTPK---NPEKTIKLIKEILSK-DPKKAQLISAFANQSIF-FNISNHLRKNVEGLAG--CLLK
CLam_WP_007557209.1  LFLDFLMLK-----HNEIFKGLKDATKSSKFVINGNDIASK---DSFDMLKSLIKIFPN-DFKKIQLISSFAQEGIFINKPFAHKIANIEHINE----TM
CLam_WP_007556843.1  MLEDFHKLG-----DSINNQLSLDLTR-STYIINGKVINKS---NPEKMIEEIKDMVHQ-DNRRLQLISTFSNQAIF-PNLSIHIMKTDPRLGQQLYGSK
CLam_AHA27935.1      LFLDFLMLKH----NEIFKGLKDATKS-SKFVINGNDIASK---DSFDMLKSLIKIFPN-DFKKIQLISSFAQEGIFINKPFAHKIANIEHINE----TM
CLeu_PTL86946.1      MFKHFRELDS----LKLDKQFKLDINR-AKYVINDKILLKE---EPEKMLSKLTQMFSK-DHKKLQIISSFANQTIF-ADPYLHLMNAENRLIE--YSSI
CLeu_PTL86738.1      LHNDYLKLQG-----KDDKALSLDIARCGEFSINGKIIPKE---EPEKMLDSVKKEL---TPEQFQLASFYATQIIT-SGSAIRMEIEIIDEFG--PISS
CLeu_PTL86945.1      FFYEFRMLE-----SDKVYKQLFDASKASEFIVNGQNIISD---NLDGILHNLRKIFPN-DFKQIQLISAFAHEGIF-DKPFTHKIPPIDPSNHFRYVAT
CLeu_PTL86771.1      FFRDFRIIKY(5)PLDRLYKKLNDAGKASEFIVDGKNITSN---SPENILNSLEKIFPN-DFDKIQLISTFAHEGIF-DKPFTHKIPPIDLINNYKHVLS
CLct_MBL0848807.1    CFQHFNKLPE----NKLFGDFLTDVTR-GEYTVNGKKFAAETNHDREILQKQFISMFPKNDLKQAQLASSYANQAIF-GEPVAAIMEKAPHLHA--QPVE
CLas_BAP26205.1      YFRDFKLLGT-----NKVYKNLLDASRATEFIIDGKKINID---SAQNMLAELNKIFPK-DFEKVQLISSYAHEHIF-CKPFTKDLLNLANKNI--YQLS
CLsp_WP_182016855.1  LHTNFQKVKMQEDLLKIDPQFRVDFKR-ANYTINGHIMSREG--DIDLRMKDVAKAFSG-NEDDIRLFSAYGNQSVL-AAPQIALINKIKNFSS--YGSS
Lsp._MBA5723779.1    LHKNFMNLRK--GLLDVNYQFLRDLSR-TKFVINGNTIAKG---PSLKILEDVKTAFSG-IEDNIRLFSAYGNQSIL-TGPQMILARKIKNFGS--YGSS
Lsp._MBA5723780.1    LHTNFQKVKMQEDLLKIDPQFRVDFKR-ANYTINGHIMSREG--DIDLRMKDVAKAFSG-NEDDIRLFSAYGNQSVL-AAPQIALINKIKNFSS--YGSS
Ssym_NIH12428.1      LQDDFHKIKVT---TQLSEQFLLDFDR-ATYKVDGKVIPRGD-------YIQFEKAIP--DIKKRQLISSYANQTSL-ADPSIGAMSFFPDSFAK-HGAH
Baph_WP_158360843.1  TYKDMYEKLEI--PEHFDKYFMIDFDS-AVFLIDGHLISST---TKEGMMEGFKKLITN-DIEQ-KFISQYANPALL-KQAYLKLMAEHPELNK--SRIV
Baph_WP_075474406.1  FQNNFNELKK---TLKLDNDFLSEGED-SVLQVNGQPISKH---SPATMLNDFKYVLP--NLESRQLISSYSHQDLF-SRPYLELFSERPELAD--LKLK
Baph_WP_158345241.1  LQNTFETLQS---KSMLHSLFLKKLGR-SSYEINGVKIPTS---SARIALKKMINIIP--NAESIQLISTYANEGVF-GQAYSNLLSKYPEVSK--LRFN
Baph_WP_075432350.1  LQNSYLRLNN---TRFLNKNFLTSLLK-KEIYINGKKIPASD--SIAETIKNIQKIIP--DYNAQQLVSTYAHPGVL-KMAEKQLLKEHPEIND--RISQ
Baph_WP_075434189.1  LQKSFKKLIQT---SVLNQIFLNNLLG-KTIYLNRRKISSN---QFEDIVHALHKSVK--DLEAQQLISTYTHPKML-DIALENLSTRFPEVEY--RESH
Baph_WP_158353850.1  YQDTYEILNK---TMQVHDTFLSNFEN-EYFQINGKDMNSS---KIQNFTKDFMQTVP--DLELQQLISVYANQNLF-NHAYLSIFLDNPELLD--FLPK
Baph_WP_075472953.1  LQKEYKNLLKT---PALDMRFLSTMDD-SYYTINGKSATNE---NPHDVLDLIRKEIP--NFQTQQLISSYTHSGLL-VEPLKQLQIRYPDLRT--EEIF
Baph_NIH16844.1      LQKKYLNLIKT---KNLSSDFDIYDST-SNFKFENHYVVYKH---SKCAILNNLKRFFP--NLEDRQLISNYVNGKVL-LNAHKIFLSEKPEFNK--FYPN
Baph_QCI26175.1      IFHDAQNCT------NIFGSNIKIPTN-NGIIIDGKYLSSE---LHYSTLDSLKQFIP--NTILRNLVLSCFQHDLT-SQPCLEIFYHSKEIEH--FKPE
Baph_WP_158342129.1  LEDEFKEIKN---SNNLSNNFTNKFKK-SEYVWNDSLLQNQ---SLDQFMRDFKEKIP--DLEDQKIFSVLASKKLF-EVPHEFIRENYPQVHS--YENQ
Baph_WP_158349688.1  IFNEAQKIK------NIRDENINFISN-N-IIIDNKIISSK---SNHKILNSFKVHIP--DIDLRNLVLSCLKQDLI-SKPCLDIFYNKVEISK--FNPE
Consensus/80%        bbppab.b.......pb..pbb.sh.p.spa.bsGp.bspp...s...hbppbbphbs..s.cpbpLbSsasppsbb.s.s...bb.pb.ph....b..p

Secondary Structure  EEEEEEEEEEE  EEEEEEEEEEEEEE                EEEEEEEEEEEE       EEEEEEEE
CLas_WP_012778668.1  GSASKFTAKTLTNGEVAFTAKYTTKVQAVDKIA-------GKPLKEYGLKISGILSPDKATELQRSFYLK
CLas_WP_012778667.1  NPRYSYQFNTLKDKTISFVAKEEGLVTYLN----------GSLHRNYGIKAEGILSRNAPPELHFSSYVN
CLaf_WP_148407341.1  NAASRYVANMLKNGTVEFTATHSANLKK-------PAEIAGPAFKEFGLKISGILSPDKATSLSYNYYLK
CLso_WP_013461761.1  KTYNSYKFDALEDGTINFSATYKGNFSPVD---------GSPSTHGYGVKVDGILSKQSIPELHFTPIIE
CLso_WP_013461760.1  NGKTTFQINTLGEEGVRLSAKYTASLVTENQAI-------KNPLREYGLKIDTILFPNKAPQFTQYFYTK
CLam_WP_023466137.1  NGKTIFEINTLDNNIMQIKATYETKTIPTFEKG(4)N---IKKLNGYGHKIELKLSPDKIISADPYFYTY
CLam_WP_007556842.1  NRKIIYKIDTMHQDTVKITAKYDADILRAKKTKIFDNSTKEIKFKKYGLKMEMTLSEDKVIEAKPYVYVS
CLam_WP_007557209.1  KGKTSYYIDTLENGELKLTAKYEGNNINN---------------KNFGVKVEGNISTNKPAKLQYSYYS-
CLam_WP_007556843.1  NGKTIFEINTLDNNIMQIKATYETKTIPTFEKG--DGYDNIKKLNGYGHKIELKLSPDKIISADPYFYT-
CLam_AHA27935.1      KGKTSYYIDTLENGELKLTAKYEGN---------------NINNKNFGVKVEGNISTNKPAKLQYSYY--
CLeu_PTL86946.1      NTNILYKIDTTDKNIVKITAKYETDLKKVDDDK(4)NIKKEKPLKSYGIRMEMTLSPEKIISAEPYFYIN
CLeu_PTL86738.1      SNNSAVQVTTLPDENLEITYQSTKKYDDSKDIN-------PKGLKSSHIEVKGIVSRDKEPELKSSYYT-
CLeu_PTL86945.1      NGKTSYKINTLANGKLAFIAKYESKFVQADGSP-------NINYNNFGVKVSTIISTNTIPEFNYSYYYN
CLeu_PTL86771.1      DGKTSYNIKTMNNGNLEFTAKYESNVIRGN--------VSPKTQNKFGVKVSTELSKNKAPELKYAYYYV
CLct_MBL0848807.1    NTHISYTMNSLDNNKIKLSALYERKVSIPDEID(4)AAKGDKYFTGYGIKIDAILSPDKAADLHYSYYL-
CLas_BAP26205.1      NPRYSYQFNTLKDKTISFVAKEEGLVTYLNGSL----------HRNYGIKAEGILSRNAPPELHFSSYVN
CLsp_WP_182016855.1  NAKTVYTVKTLDKGRLEFIAQHTADLRHIDEFK-------TDGLKSYGLQVKTILSQKEAPKLEYSYYV-
Lsp._MBA5723779.1    NAKTVYTAKTLDKGRLEFIAQYTADLHHTDKLK-------TDGLKSYGVQVKAILSQKKVPKLKYSYYAH
Lsp._MBA5723780.1    NAKTVYTVKTLDKGRLEFIAQHTADLRHIDEFK-------TDGLKSYGLQVKTILSQKEAPKLEYSYYVR
Ssym_NIH12428.1      NSNVSYEIWNIPDSKIKLIAKVESKLTPVDLAD------GEKIYSSYGLKAEITLSENTPPKYKYSYYLF
Baph_WP_158360843.1  RAKNYYEITHLEDGTIRIIATNLSEFYSIDAND-------VTHHHSYGVKSSVIFSYTKAPIIKHSYVIN
Baph_WP_075474406.1  DIKVSYVADELDDNQIQLVATSKARLDSNTSYK----ADGNTYDDSFGIQASMILSKDKVPNIEYVYFL-
Baph_WP_158345241.1  NSHVSYKINALDKDTFRIVATNLSELKPTYDFM-------QTPYHSLGMRSSMIFSKKNHPVMKNAFFV-
Baph_WP_075432350.1  NMHITYRIDQVDNESFRISILKHAYLRPSMFHS-------PNDIHAYGLRANMLISKTSRPKLQFTYF--
Baph_WP_075434189.1  NERIVYEIDKINSDTYKVAVTKFADLKPSNFSD------DMSEINQYGIRSTMIINKNVNPEVRYAFFVS
Baph_WP_158353850.1  HPVFHYKIDIIDPQTIKIVGVNTGILEKKYSNN-------QLKTVLFGVKTNVIVSKNDAPKIEYSYFI-
Baph_WP_075472953.1  DMFTSYNIRKSDHDKVHITITRTVDIRPPEDEL---ENFDIEKLQSYGLRTDMTLTKYANPTLKYSYFVS
Baph_NIH16844.1      NSHMNYVITKLPDGYIRLIVMYFTHLGQLDDNSD------SSDYSRFGMRAVFILSKDHLPNVKYTYF--
Baph_QCI26175.1      KAAVFYVIQTNQDKTITLTVTYLGRLIKLVARKT------SQQYTNFGARASIILSIDELPKIQYFNFIN
Baph_WP_158342129.1  DPIYRYEFIKSENNEHQVIATKIIQSNKLEKQG-------SSEKNMFGVRTIVTFDRNRISDIEY-----
Baph_WP_158349688.1  RSAIFYSIENLKHRKIKLTATYLGHFIKDETKEN------NITYKNFGARTSVILSKNKLPEITYFNFIN
Consensus/80%        ps.h.Yphppbpp.pbpbhspb.sph.................bp.aGhchphhbS.pp.spbpb.hah.
```

HHH → alpha helix

EEE → beta strand

Supplementary Figure 5. Multiple sequence alignment (MSA) of the beta-sandwich domain associated with the YdjM domain. For coloring themes refer to the Supplementary Figure 3 legend.

```
Secondary Structure   EEEEEEEEE     EEEEEEEE     EEEEEEEE      EEEEEEEE         HHHHHHHHHH   HHHHHH   EEEEEEEE      EEEEE
Tfra_WP_051965836.1  IDALSLVPT--LNFSKWDFIVEKK---EKYYTGRAS-LP--STLQVEGVYYKN---SVPPGLLNIALQAE-PIQAFQDFARHPMITYKTCS--EGHEVKF
Ssil_WP_094604719.1  LTRLLVMPSL-KSVFSWDFVVETK---DQYHVGQVGLLQ--QFLTIHTKFTKQ----TLSPATQEAHKTV-LGEFFSSFTPFSYFTEYQDNEHAGKHVHI
Opfe_WP_054876990.1  IRKIHLLPSM-IAFYKFQYIIEGS---MSKIVGDINFLY--HRVRVFDIFKGQ-----DDGIEEHVLKSR-TAKYFKEFTPIFHIDFEKIN--DGYKALL
Nbac_MBI5406191.1    SDSVAALPQP-FSPFKWVLLIDTG-DTLYQSFIDIDIKG-NDRHDKINMIWKK---WPDSPWVQKARKLP-GVGFYLWFARFPVAMVEETP-DGHHLVEF
Nbac_MBI4823699.1    VCKLCPLP---NDFLRWWFVAKSN---DGIQVGFADLFT--KRICIQETYPSQ----ADEPAIERSKDDK-VVRSFLYFAKYPYAEVKKQN--GKTVVTW
Ntru_OWZ84931.1      INKMVVLPAS-KSFFHWDFIVETD---YIVYVGQAPIFR--RAVRIKEKLSKRIEEEIENSLINKALNSK-IGKLFQEFTPHYLVKHHKTS-DNKHLITF
Maby_GGE26688.1      PGHYTLVPT--IRWNTWSLVLEEK---DRVRMGEW--LG--GQIVWTQVLDKR---ALEHPAVKASRKDS-KIQAFLYFSDYAYPQVKETP--TGYEVRW
Fbac_PKM87113.1      DSKLVILP---LRFSVWDFIASLP---DKKVVGTVDSIK--RKVRILGCFDN-----IRNHLGQAVLASV-LGKYFVDFTPYYHISCEASE--GKYVARL
Fbac_NPV27839.1      LKRVSVLPPV-VGISNWNFIVETE---NSMFLGNVDLNR---GVTTREVLPKP----QVDPVVTAAMRAP-AAEVFLGFARHPYLTYEKLG--DRYLVKI
Dlac_WP_073029145.1  KDQVKVIPAM-YHPFTWNFILFQE---EHVSFGVIRN----QIPSICRALPKL---TEDNPSVTNALEGN-VAEIFSQFTPHYHVIAHYLD-NEECRVEF
Dalk_WP_031517982.1  AGAYSVVPT--LGINFWHCVVEKR(5)KKILVGHIS-TA--GRVVVEREFIVN----PPRYIMDSLKKSE-LAQQFISFARHLAVWINKKA--DGYEIVL
Dbac_MBI5328482.1    SSNIEAIPLP-LSPFRWSVFVEDD---ERFYQVNTDILK--YNLTINSFEKKP---QANNNIIEKVQNLE-IVKIYLWFARFPVVTVKEQD--SGYLVEY
Cend_RKP58239.1      SSQVLIFPT--FSLFNWSIVLVGH---DAVQAGVWRY----GNIIWQSPINLG---EAGDRYVKCSEQTD-AVKLLKNFSPYLAVQKITHP--DGIKIRW
Cbac_PLX31380.1      IISIDVLPAL-MAFHKWNFVLSTN---SHNIAGQVNILN--KRIETRKKLRRE-----DKDLVKVFKGSN-AGQYFTNLSPNYHVTMKSDE--GRIILEG
Cbac_NLI60421.1      NQRIVVMPSM-LRLFKWDFILYQE---KRIITGRVSILT--KDVEVRDYLER-----SHSKLENVVMDTK-IGRFFKEFSPHYHVEWTKDG--DNHKAVI
Cbac_HHZ17469.1      PFRIYVLPSGRMSFLRWDFIVKST---LKDLVGSIDLGS--GRWRVFRELDHP---PEEVREIQEILPLTPLGQTFGEFTPLYHIKCEKLG--DKFICHF
Cbac_NLT94498.1      DGRITLLPPL-LGLRKWNFIVERC---GTYYLGQVDSFS--GEITIKEELFPE----EECPSVLASKEDP-EVKIFLDFARFPWYSIEKQD--QDTVIKW
Cfer_WP_028051626.1  EAKITVYPDP-LDFFSWRYIAQEG---KTIHQGEAKFLS--GKIIETKTLESS----EIPEESRVALSTP-EAKAFLNFAKFTYVKVKDTP--EGRKIYL
Bace_AYO29893.1      LHRVLVMPSM-FGIFSWIFIVETK---KSLLVGSINSFPKKLFKPVLKELLKC----KDHPMVQLALNSK-PGEIFKSFTPFYYIDLIRKG--EYFYVQF
Pant_WP_138192560.1  GDEYTAIPTV--NWSVWNVVRKNRE--GNYTIGVWRSG----RLKWTDEYVCE-----SSPLIEKSKRHP-AVASFLSFSSHPCPHEERRG--SETIVRW
Consensus/80%        ..ph.hbPs..bsb.pWpbhhppp....p.bhGphp.h.....h.h.p.b.p.......p.hhp.sbps...sphF..Fs.a.hhphppps....bbhpb

Secondary Structure  EEEEEE      EEEEEEEEE    EEEEEEEE
Tfra_WP_051965836.1  TDLRYRFRD-YHPFFATVTLNQNLQITGACLGK
Ssil_WP_094604719.1  YDLRYFFNQ-DFLHSATIIFDCNQHPCDSYIKT
Opfe_WP_054876990.1  TDLRYILKG-EFLHHATIIYDEKMNILEEKFNP
Nbac_MBI5406191.1    IDLRFDILRGRIPFTHRVEFNEGGLVIMEKFTT
Nbac_MBI4823699.1    KELSFSFIY-GEHFVAKVFMDENGKVLSSYFKF
Ntru_OWZ84931.1      IDLRYHIGE-RFLHHATVKCHNQGMIEEELFQP
Maby_GGE26688.1      VDVRYHTKK-HFPFQAIALLDHHCRVIESHVGW
Fbac_PKM87113.1      MDLRYRVKD-RFLHNATLVMDSDLKVEEAIFQP
Fbac_NPV27839.1      VDMRYQVRN-RSPFMVVVQLDRNLNVIDSGLGS
Dlac_WP_073029145.1  LDLRYWVKG-DFLYTGNVYLDLNGEISHEIFHP
Dalk_WP_031517982.1  GDLRYRHRQ-QYPFAAYIKLSNNLEVLDEDLRY
Dbac_MBI5328482.1    FDLRFNALPPRKPFLLSLFLDKNGSLNHAELMF
Cend_RKP58239.1      LDLRYLERA-NYCLMACVLIDQSYTIQRSYIGW
Cbac_PLX31380.1      IDLRYFMRD-NFMHHATVVIDENLNIVESFLQP
Cbac_NLI60421.1      TDLRYYVKG-KYLHRATALFDAKLKPVHAAFQP
Cbac_HHZ17469.1      MDLRYRVEN-RFMHNGVLVLNENREVEKAFFLP
Cbac_NLT94498.1      SDLRYKLRE-KEHFTLTAKSKEEILWKAN----
Cfer_WP_028051626.1  LEPRYHFRG-HYVFGVLIELDRQNKVVKTYLNQ
Bace_AYO29893.1      YDLRYLLKD-RFLHSATVIVSEEGDLLEGLFHP
Pant_WP_138192560.1  LDARYQHRK-QYPFLAVVKYNANKEPVFSYVGW
Consensus/80%        hDLRYbhp..pb.a.shhbbspphph.p..b..
```

HHH → alpha helix

EEE → beta strand

Supplementary Figure 6. Multiple sequence alignment (MSA) of the Exonuclease-Endonuclease-Phosphatase (EEP) (PF03372) family. For coloring themes refer to the Supplementary Figure 3 legend. The sequence, CLas_WP_015452829.1, consist of two protein sequences, WP_015452829.1 and WP_015452830.1, which originated from the same gene in *C.* Liberibacter asiaticus.

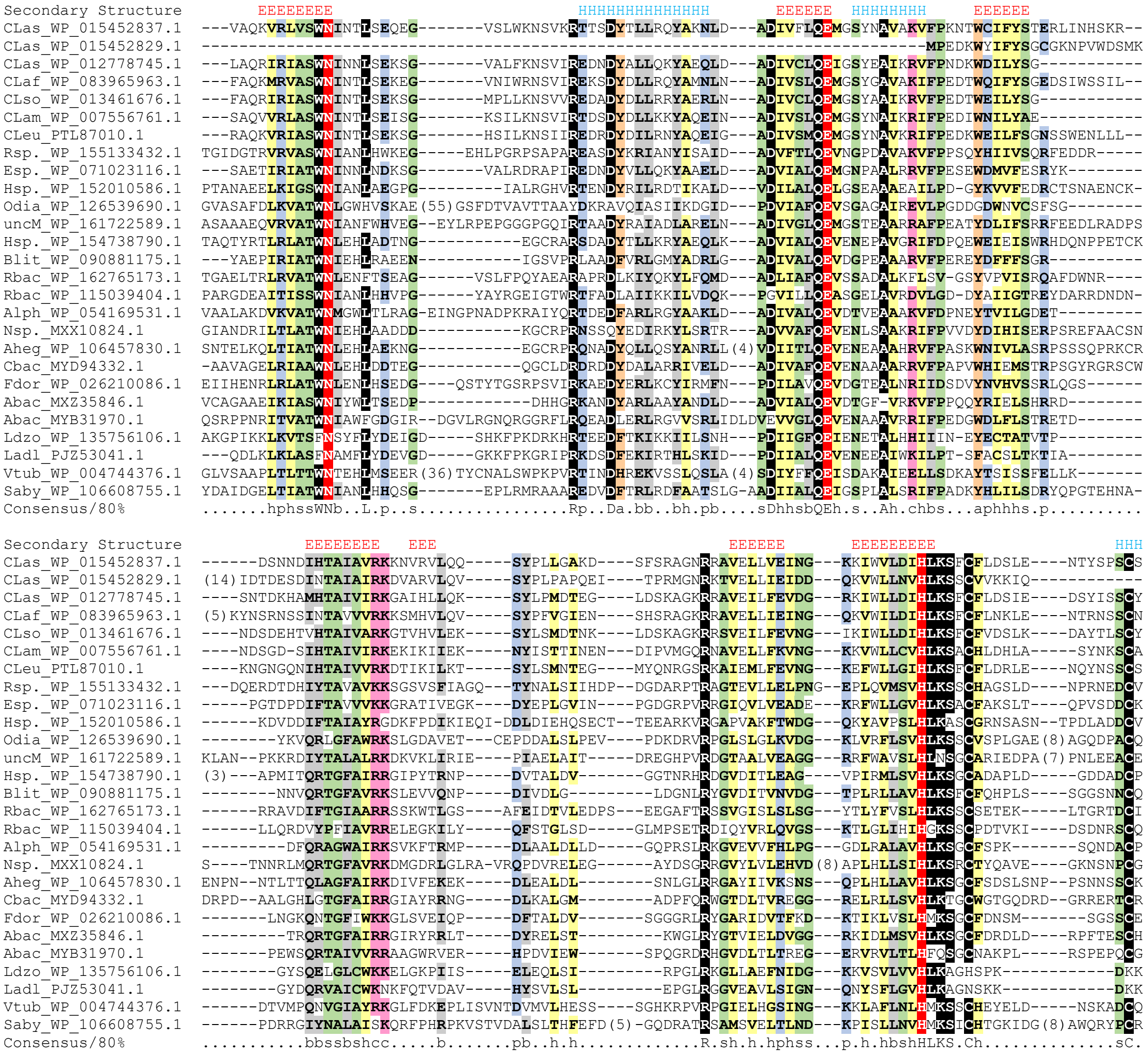

```
Secondary Structure    HHHHHHHHHHHHHHHHHHHH  EEEE                                HHHHHHH                                                               EEEEEE  HHH
CLas_WP_015452837.1    LLSQQAQWLKDWITQKKESLVPFVIAGDFNRKINYLGNN----DDFWKTIDPNDSL-------IRFPKEKDSRCN----ANKNLRNKIPIDYFVMDQNAY
CLas_WP_015452829.1    MLSQQGEWLKKWADQKIKTGIPFVIAGDFNRKINSIGDT----DDFWQKMDPDGLL-------IRFPQEKESTCN------VIKRNKSSLDYFVIDRDNK
CLas_WP_012778745.1    MLNLQATWLKQWVDQKNNLNMPFIIAGDFNRKINHSHSG--IKDELWQKINQDNTL-------MRLPHKKN-----------------------------
CLaf_WP_083965963.1    LLNKQSKWLKLWIDQKKETGIPFIIAGDFNRKINYLEDK----DSFWQKINPDNSL-------IRFPQQKETSCN----ANKNLLNKTPIDYFIMDKNAY
CLso_WP_013461676.1    TLNLQVNWLNKWIHQKKRSNIPFIIAGDFNRKINHFGDN----DELWGKISKDTIL-------IRVPNKKRSWCN----AHKSIRKREPIDFFVMDQNAY
CLam_WP_007556761.1    ILNKQAIWLNRWINKRKNENMPFIISGDFNRKINRYGED----DEFWQQISNNTNL-------IRFPYYKRSKCQ-----ANHYKNSEPIDFFIMNKLAY
CLeu_PTL87010.1        ILKLQIDWLKEWINKKKASNTPFIIAGDFNRKMNNSGNN----DEAWLEINQNNDL-------IRYPSYKRSKCR----TASKYKNSEPIDFFVMNQIAY
Rsp._WP_155133432.1    TLANQRAPLEAWIDELTEDNTPFIIAGDWNRRIDRFAQN----DHLWGEIDDNSPAP---LDLARFPEGITSPCL----AGTSGHFPDPIDFIVLDEQAL
Esp._WP_071023116.1    TLAMQMKPLEDWIDEKEATGLPVVIAGDFNRKFDVHGQN----DHLWAEIDDADPASLDL---IRTPFREASRCP----TTRESDRPEPIDFIVTNKLAT
Hsp._WP_152010586.1    TQAKQIDRLIEWIGEPEQAGYRFILTGDWNRELGTSS------DTIRKRLEAVDANV--------RFEPQERVCW(28)WAYTPDSGNDIDFFVVLGVPS
Odia_WP_126539690.1    VLQQQVAPLEAWIERKAAGAAAIVVLGDFNRNLPHEIAA(21)ASRIGEVDDGEPAA(6)EAHCPVTPDAEAACA(17)ENLGCRNPIGLDHILVGSGVL
uncM_WP_161722589.1    MLVRQIPILERWIDVRERAGEDFVLLGDFNRVFDRAN------EPLWRDLDDRDPQT---LDLFMVPYRQALVCS-----GHSPEPDRSIAYVILNKRLW
Hsp._WP_154738790.1    PLRDQSKVLNSWIEARRKDGLPFVLLGDFNRRLQNEE------EVVGLLGVRSGLT-------LSVSREAVSRCH--------AWTDKFIDHIIFDQKSK
Blit_WP_090881175.1    RLKQQIPVLEDWIDARAIEGVPFAVLGDFNRRFEIDENLPSE-DSFWQDIDDGKPKGLDL---SRVTAGRTSECW-------GGRHPDFIDHIVLDQIAV
Rbac_WP_162765173.1    LLRAQAEIINNWLEKNIKAGASIILAGDFNRNIFRFSEDPFT-SELLRAVRSSGTKY------RIVPSSGVTGCP---------TRKFRIDYIIAVGEIV
Rbac_WP_115039404.1    LIYKQFLSIGDLIKKEAGQDDAVLVVGDFNRELLNPR------LLEWRKTYAPWAAS----------LIAPPACI-------LRPSLQPIDFVIVSKLPT
Alph_WP_054169531.1    VIAEQMPILSKWIDARQAEGTSLLVAGDFNRRLRDE-------DPLWKVLDNGPSPL------KLATAGKANHCW-------AGEYPEFIDQIILGGPVV
Nsp._MXX10824.1        ILNKQINVLSAWINARDRSGQNFIIAGDFNRQLDQLS------DAMWLRLETGGKPG---TYVDLEKALHGVKHP----KPYNRKYPYAIDHVIYNQALD
Aheg_WP_106457830.1    ELATQLPILEKWIDERVKKGERFAIAGDLNRRLNIPG------DEFWQEIDDSEPDI--GADLETITEGRLSSCN---------RYKDYIDHIVLDKGVT
Cbac_MYD94332.1        TLRAQVNHLKAWADARREEGAAFVILGDFNRRLALPG------DWAWGVLSPPAAPL------RLLTQGVAFRCD--------PRYPAFIDHLVAGGGAT
Fdor_WP_026210086.1    TLMDQIPVLESWIDTAATKDEPFILLGDFNRRFNLDS------DRVWSEIDDAEPAN---ADLTTVTLNMPLSCR-------DNRYTEYIDHIVFDKKSF
Abac_MXZ35846.1        ELKSQVRPLESWIDRRLERGRSFVLLGDFNRRMDRRG------DDLWRFIADGNPKP-----VYRVNAGKRPKCW-------GSRFPEFIDHIVVGPIAG
Abac_MYB31970.1        FLRMQFAVLKAWLFERMAEEIPTVVAGDWNRFLSRPA------DEAAGLPGEAL--------VLPPAGSAPACW-------DGFFDHYVDHIVAFAPRG
Ldzo_WP_135756106.1    ERKEQILALKDILPSL----GKFVLLGDFNEVLERR-------VDLLKILQRNLKL-------KVANYKQKSDCW--------QHNDGFIDYLITNMDWQ
Ladl_PJZ53041.1        LRSRQLDVLNKILKLK----KNYFLLGDMNSPLGKD-------NRSWNLLSKGLDL-------KNPGRYTKQTCW---------GHESLIDFILTDLSIS
Vtub_WP_004744376.1    TYAKQVVKIEEWIEKMTEDGIDFILLGDFNRVFKVDW--------SYDARSDGSDAQ------SSPTKDSKTRSF(23)KFSDWNCYEKIDRFFLSKAFA
Saby_WP_106608755.1    TLAAQVEILENWIDMQGDRGRNVVILGDFNRQLNATDGTGASADDVWRDLNDGKGAP-----LVKGPSDPDDVCW----PNHDGRHVSHIDMIIANDRLV
Consensus/80%          hb..Qh..Lp.Wbp.p...s.sbhbhGDFNRpb.........s.hb..bs.s.............s......C...............IDabhhs....

Secondary Structure    HHHH           EEEEE                    EEEEE
CLas_WP_015452837.1    KFLIQES----FSEILYNEDDIKS---RGKRLSDHCPISIDYDF
CLas_WP_015452829.1    NFLIDNS----FSIVSYDQSDLDT---RRSKLSTHCPLTIEYDF
CLas_WP_012778745.1    --------------------------------------------
CLaf_WP_083965963.1    KLLIKNS----FSEVLYDSNDIQI---KGNRLSDHCPITIDYDV
CLso_WP_013461676.1    KYLIENS----YSEVSYIEEDIKK---RGYRLSDHCPITTDYNF
CLam_WP_007556761.1    QYHVEGS----FYEFSYRNQDIKE---RRYNLSDHCPIIIEYDF
CLeu_PTL87010.1        RYFIKGS----FSEFLYRDQDIKE---IGRRLSDHCPIIIDYDF
Rsp._WP_155133432.1    RWADRDS----FQIVDFKPNDRP----HRKKISDHCALYIDLVI
Esp._WP_071023116.1    EAIIDQS----FVELLYDETEAAE---LGNRLSDHCPKSIDFKF
Hsp._WP_152010586.1    DIQISAE(32)NKVLVFSP----------ANPSDHCPIRMTLGS
Odia_WP_126539690.1    SDGANKV(5)-GRSLPADQNHLD----PLLAVSDHCPLRAVVRF
uncM_WP_161722589.1    AWAEPSD----SPKLDV----------TGKNVSDHCPVFLDLRL
Hsp._WP_154738790.1    AFAEFTH----FAELKFAEP-------EAKYPSDHCPVSVDVTV
Blit_WP_090881175.1    GWVSSGS----FEQLVYDEAD-EQ---FDSVLSDHCPISVVLDI
Rbac_WP_162765173.1    DAIIESS----VREYIPTEYERI----FGAKVSDHCGMSVDFLL
Rbac_WP_115039404.1    GGTIASG---STLSNLLPTLD------HPARISDHCPLSIRVDF
Alph_WP_054169531.1    RRAKEGS----FGVLVYTETSRD----DKKRVSDHCPVSIEIR-
Nsp._MXX10824.1        GLIV--------EAKTSFDA-------KGGKYSDHLPLFAVFDL
Aheg_WP_106457830.1    RAVSERS----FFQLVYKEA-------ESEHPSDHCPIGVTIDM
Cbac_MYD94332.1        VVPGS------FREMP-----------RRGPHPDHCAVYADFRL
Fdor_WP_026210086.1    EWVDLSS----FRQVNYRQADKA----DWDKISDHCPVVIEIWV
Abac_MXZ35846.1        KAVKPNS----FEELTYSERNYFG---WRKRLSDHCPISIRLVF
Abac_MYB31970.1        REIAPRA----FEEVRYRA-PRS----ARDRLSDHCPLVATLGF
Ldzo_WP_135756106.1    QGS--------FVQTKFESDDGNF(5)AEKGLSDHCPVSADLLL
Ladl_PJZ53041.1        NAD--------FRQFPFPEDDGEF(5)SERGLSDHCPVVLEIAL
Vtub_WP_004744376.1    ETLIWKT(5)EAHGKNYPN-FSK----DKVKPSDHCPITLTLPL
Saby_WP_106608755.1    SEAQMST----ARKIGYDAYDDPT(8)GRYRLSDHCPVILTLGG
Consensus/80%          .hh........h..h.h..............hSDHCPh.hpb.h
```

HHH → alpha helix

EEE → beta strand

Supplementary Figure 7. Phylogenetic relationships of the Liberibacter EEP related sequences and other known EEP enzymes.

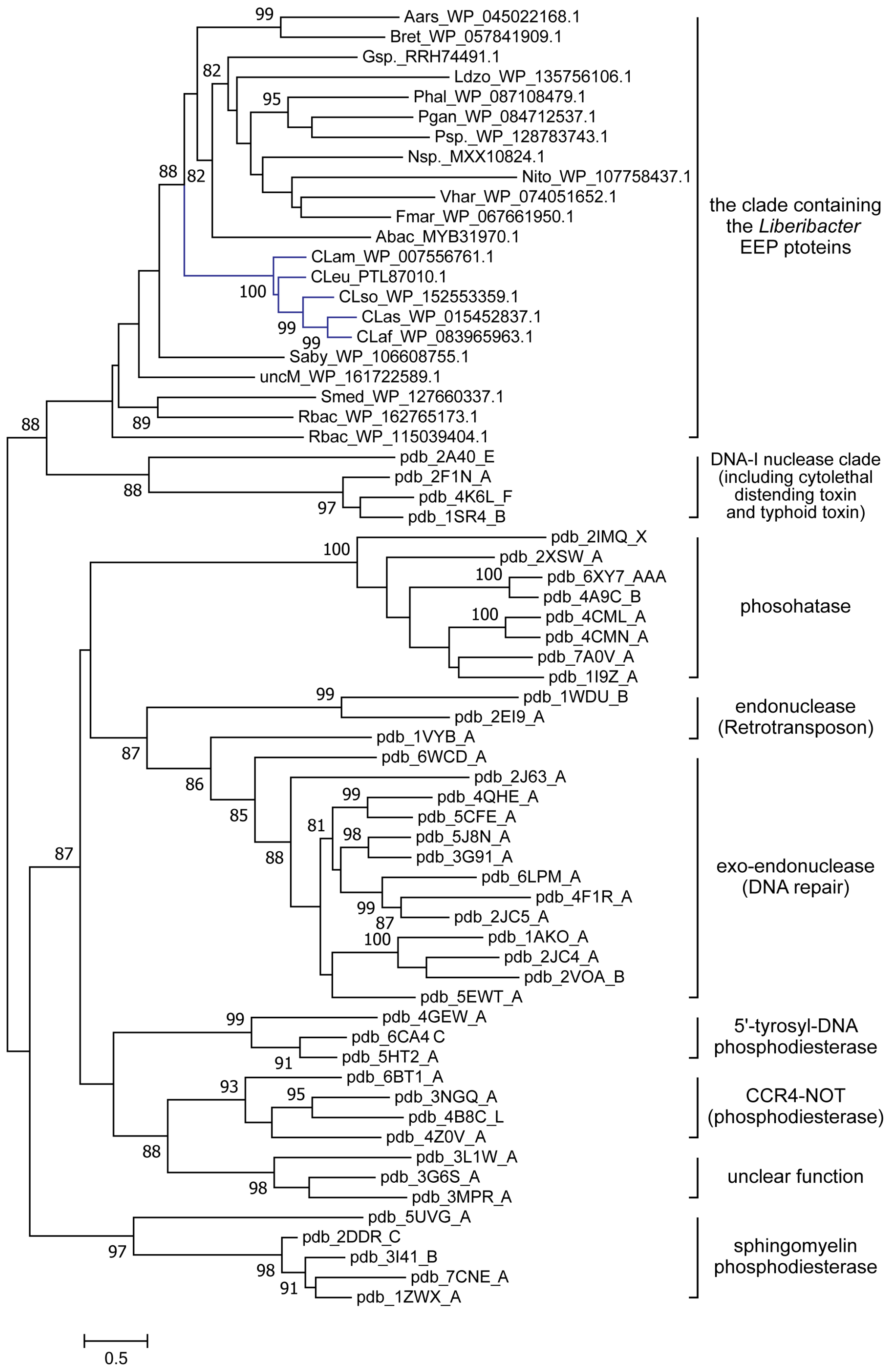

Supplementary Figure 8. Multiple sequence alignment (MSA) of the potential transporter (which is wrongly annotated as Peptidase_M75, PF09375). For coloring themes refer to the Supplementary Figure 3 legend.

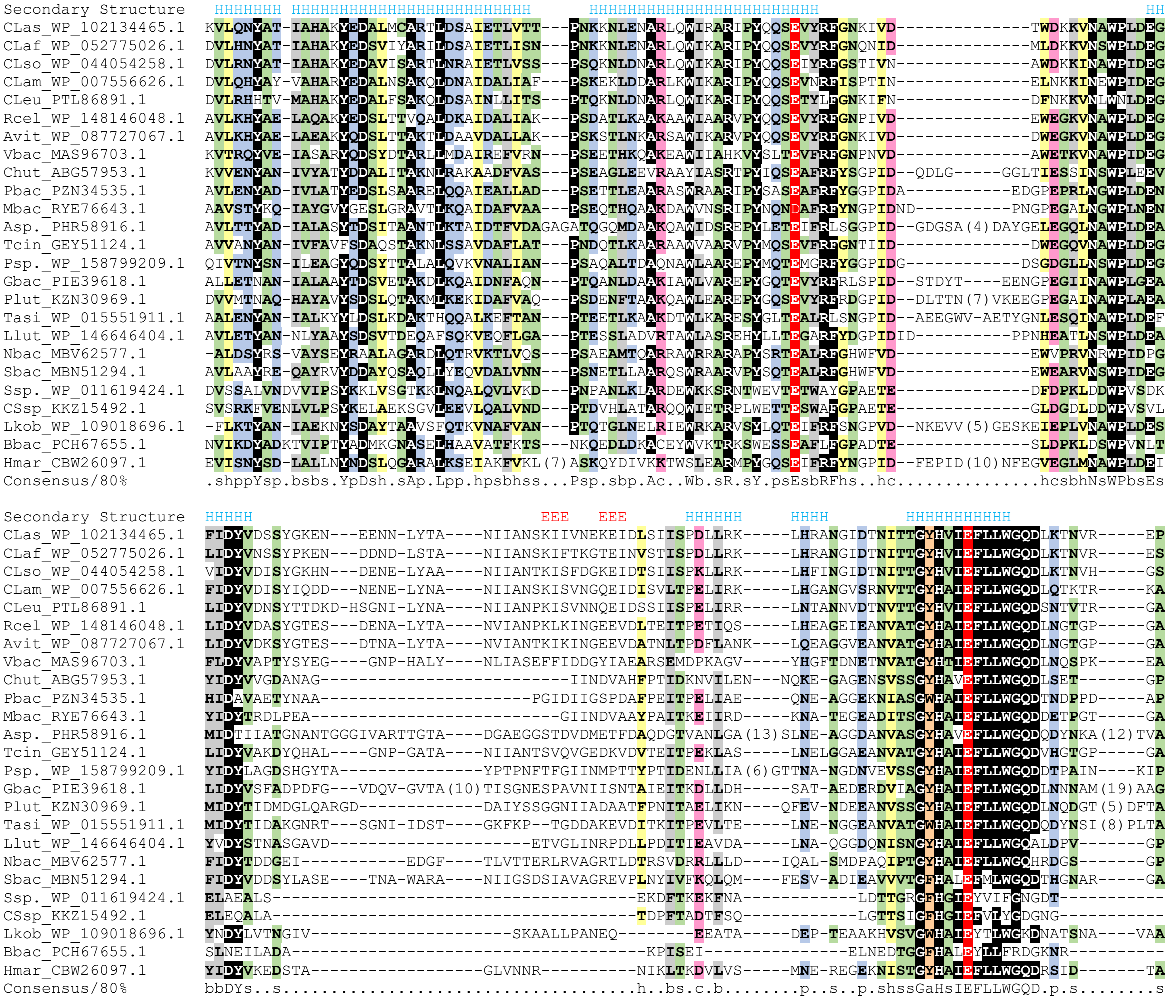

```
Secondary Structure                                HHHHHHHHHHHHHHHHHHHHHHHHHH           HHHHHH           HHHHHHHHHHHHHHH HHHHHHHHHHHHH
CLas_WP_102134465.1   GNRPYTDFDIGNC-----TGGHCRRRVEYLKVVSKILVSDLEEMMKAWGPDG------QATKDLM----KDINAGLNSIITGMTSLSYNELAGERMNLGL
CLaf_WP_052775026.1   GTRPYTDFDIKNC-----TGGHCQRRIEYLKVVSKMLVSDLEEMMKAWEPNG------EATKNLM----QDIDAGLNSIITGMTSLSYNELAGERMNLSL
CLso_WP_044054258.1   GERPYTDFDIKKC-----TGGNCQRRIEYLKVVSKILVSDLEELMKVWKPDG------EETLNIM----KDTNLGLDTIITGMTSLSYNELAGERMNLGL
CLam_WP_007556626.1   GDRPYTDFSIKKC-----TGGNCKKRADYLKIASTLLISDLEKMAKSWSAEG------ETTKLLM----KDIKSGLNLMIKGMTYMSYGELAGDRMNLGL
CLeu_PTL86891.1       GNRNYTDFSIKQC-----TGGNCKRRADYLKAASKLLVSDLEEIKKSWENNG------ESTKIIM----KDIKSGLNLMISGMTSLSYSELAVDRMNLGL
Rcel_WP_148146048.1   GNRPYTDYDKANC-----TGGNCDRRAAYLKAASTLLVADLGDMVKAWAPDG------DATKNVE----ADGNAGLTAILTGMGSLSYGELAGERMKLGL
Avit_WP_087727067.1   GNRPFTDYDLKNC-----TGGHCDRRAQYLKSASTLLVSDLQEMVDAWKPEG------EATKTLL----ADPTSGLKAILTGMGSLSYGELAGERMKLGL
Vbac_MAS96703.1       GLRPYTDYVLGEN----GTGGNMDRRRQYLMAISGILLQDLTTAIQDWKTDRP---FLYSKVFFD----LPVEQQLDRMVMGMGSLSNGELAGERIQVAL
Chut_ABG57953.1       GDRKYTDYLVNSD(3)---NLNQARRGQYLLACIDLIIDNLQTLKEAWKSGNA---SNYRAGFI-----ADPKGSIGNFLKGVIGYADDELSVERMQVAL
Pbac_PZN34535.1       GRRPYTDFIPDGD----PATDRAARRGQYLRAATTLLISDLESLVDAWRKLDV(9)DNYRATFVS----GDVYTGLKRMFTGIAALSEVEMAGERMEVPL
Mbac_RYE76643.1       GKRPYTDFVEGAG----GTAKNQARRKQYLVAAAELIVEQLTTVAQAWDPKVA---GNYAADFGV(8)NDGLKDAIANAIRGMGSLAKAELSGERMTVAI
Asp._PHR58916.1       GQRPLTDYTNGS--------GEATRRGAYLKAAANKIIADLTMVRSAWLTTTS(9)GCYRAALLG(10)IDATTALKDIMTGMGVFVSSELSAERISASV
Tcin_GEY51124.1       GERPASDYVEGAG----GTGGHNDRRRAYLKAATDLLVSDLDEMVNNWKPGVA---DNYRATLEA----EPAESGLRKMLFGMGSLSLGELAGERMKVAL
Psp._WP_158799209.1   GQRSYKDYLTTAA----ATAPNGARRAQYLQIVTQLVVDNLKTMVDAWAPNA----TNYRKTFQ-----AAPATAIANMFNGIGRLAKGEMAGERMGVPL
Gbac_PIE39618.1       GQRPLTDFTTA---------PFADRRLAYLQVAAEKLVEDLESVRDGWLDNVE---GNYRDKFTNFTSRSEAVQRITEILTGMGTLSEGELAGERMQIAY
Plut_KZN30969.1       GHRPVTDFYTTTN(16)SADTVCERRGMYLKAAAELLVADLQGVVDAWTPGSG---FHYIDYTKE----ENANANLAAMLESMGRLGHGELAGERMSVAV
Tasi_WP_015551911.1   GQRPLTDYTED---------KFKERRKDYLNATAEKLVNDLQTLVDAWSTERDGAKGLYRQALLG(10)IKTDEAIKQILSGLGVFIKSELANERIAVAV
Llut_WP_146646404.1   GKRPATDYVTGGP------RKNADRRAVYLRVAVSGVLKHLTAVRDAWAPSA-----EYRTSFV-----GGGMASVGLIITGLGKMSKGELAGQRMNAAY
Nbac_MBV62577.1       GQRPWTDYALDSS(9)--PTRHCRRRGQWLQAMTELLVWELERMAAVWADKPG----TYGYRLVH----GDPELGLQRALIGLVTVSSDILADRRIETPL
Sbac_MBN51294.1       GERPWTDFSLE--(10)-SEKPCRKRGEFLKTVSVLLMRHLQEMREHWGAGEG----TPGDRLVR----GDIDTGLNRMIFGLSALSAEELAGERMQVAL
Ssp._WP_011619424.1   -PVEASD---------------LTTQQLAYLRIAGENLKSNAKQLLAAWSGPEG----FGKADVE-----ADPAKTVNDILEGMAGCL-DEVSNTKLGEAL
CSsp_KKZ15492.1       -RPTAAD---------------LTPQQTAYLALAGEAFHDTAERLLSSWTGANG-----FGSELM-----SDPGGTVLEMIEGAIGTL-EEVHLEKLGAVL
Lkob_WP_109018696.1   GQRPLADFVSN---------ANFANRKTYLKSSTDLLVDHITILKKQWSSTDS---NSYYYKFTK----DADGKSLDSVLRGIAKFAYGEWGGERMSGA-
Bbac_PCH67655.1       ---KVADF---------------SGDREIEYLEKVAENMKSNAALLHDQWVSTY------KAEFLA-----FKQSTAIDLIIDGMAGIA-DEVGAIKIAGPY
Hmar_CBW26097.1       GQRPYTDYVDGGT------AKNQDRRREYLSLLIDLLDDHLSTVTNQWQVGEK----NYRTEFLK----RDSNSVLTDIFTSMISMAGDELKSERIENAF
Consensus/80%         GpRshsDa.............h.RR..YLphsspbbhppLpph.psW.s........b...hh......s...sb..bbpGbssbs.sEbsscRbphsb

Secondary Structure                HHHHHHH     HHHHHHHHHHHHHHH                       HHHHHHH   HHHHHHHHHHHHH   HHHHHHHHHHHHH     HHHHH
CLas_WP_102134465.1   ILH---DPEQEIDCFSDNTYASYLNDVIGIISSYTGEYIRMNGEKI-HGASIHDLISHN-NRNLAQEINDKFSN---TMKDFHILKDRAENIES-FDQMI
CLaf_WP_052775026.1   ILH---DPKQEIDGFSDNTHASYINNVIGIISSYTGEYIRMSGEKI-RGPSIADLISHS-NKNLSQEIRDKFSN---TIKDFYILKNRAEYLEN-YDQMI
CLso_WP_044054258.1   ILH---DPKQELDCFSDNTYASYLNNVIGILSSYTGEYTRINGEEI-HGPSIHDLIKNK-NKNLSAEIKNKFAH---TMKDFYALAERAEHIES-YDQMI
CLam_WP_007556626.1   VLH---DPEQEIDRFSDNTHASYLNNIIGIASSYTGEYTRINSEKI-SGASISDLIRNN-YKNLDIEINNKLYD---TLKDAYIMAEHASHVES-YDQMI
CLeu_PTL86891.1       ILH---DPKQEIDRFSDNTHVSYLYNVIGIVSNYTGEYTRINGEKI-HGPSLSYLIGSK-YKNLDLEIKNKLSN---TLKSAHILAEHAEHVES-YDQMI
Rcel_WP_148146048.1   LLH---DPEEEHDCFSDNTYASHLNDAIGIKSAYTGEYTRVDGTKM-KGPSLSDLVAAK-DKALDKEIKGKLDA---TLAAMNVMKDRAQKVEA-YDQMI
Avit_WP_087727067.1   LLH---DPEEEHDCFSDNTHNSHYYDALGIRSAYTGKYTRVDGTKM-TGPSLSDLVATK-DKALDKEMKSKLNT---TMKAMTALVKRAKTVEH-YDQMI
Vbac_MAS96703.1       LAS---DQEEEQSCFSDTTHIAIYQNALSVQSLYHGQHHMHNGQKI-EGPSLSELVAKL-DQNLDAELTQKFVE---TMEAAQGVVDRAEKIEP-FDRMI
Chut_ABG57953.1       DTE(4)RQEQEQSCFSDQTYNDIILGQKGIKNVYVGEYVRIDGTIV-SGMSVSDLVKAA-DPKLDSTVRAKIYD---TDIKVAAIHAP-------FDNEI
Pbac_PZN34535.1       LSG---DQEEEQNCFSDTTIEDLRANAAGIEHVYFGRYTRLDGSVV-EGPSLAQLVREA-DPRVADEMQARMAR---VREALAAIQSP-------FDLEI
Mbac_RYE76643.1       KNR---SQEDEHSCFSDTTWYDLHGNALSIQNVWVGQYRGK-----KVGAGISDVVAKV-DPALAAQVTTDIAK---AVDILAAFEAG-NAAVP-FDVVA
Asp._PHR58916.1       DTA---SEEDEHSCFSDNTHRDIAQDIKGFVNTLKGQYTALNPADNIDAATTTSFYDLV-SDATKANIDAALTS---IDTKVSTIDNLAKTTKH-FDYQI
Tcin_GEY51124.1       EAN---SSEDEHDCFSDNTHNSHFYNGLGIRNVYLGEYTRVDGSKV-TGPSLSSLVAKA-DAATDQTVRADLDA---TQAKLQVIVDHANKGEH-FDQLI
Psp._WP_158799209.1   ALH---AQEQEHSCFSDNTHRDLYLDMQGIVNAYNGKYTTISGTVI-SGLGLSDYVKSL-NPADDTKMKTLLTN---AQTSLQGIET----NKP-FDYLI
Gbac_PIE39618.1       SSN---SQEDEHSCFSDNTHRDVVLNAQGVYNSFLGEYAGYDSD----------LDGTV-DATANAVEGYGFDD---YAAELNIEGLTAISNE--LGSRL
Plut_KZN30969.1       RTD---SQEDEHSCFSDNTHRDILLNAQGIENHYLGTYTRTNGEVL-KGAGVHDLLMSAGHKELAGELQTAIDA---TMAAVRVIDDNAKNNGISFDVQI
Tasi_WP_015551911.1   LTP---SEEDEHSCFADNTHRDIDQNFRGWLDLLKGQTNGK-----SSGVSFYDSLGKE-NKAKIDKLIADIQR---RISVMNEVAV---KEMH-FDYQI
Llut_WP_146646404.1   ETK---SRRDQHDCFSSQTLVDYERDARGIQGMYLGKYGTT------DGPGLDELVRAK-DAALDARIQKQLQT---SIDAIVAVPKP-------FEASV
Nbac_MBV62577.1       QSR---RPEQAQDQFSDNTHASLLNTALGIEQFYYGRIDGK-----AAPVALADLARHA-DANLAGQIDRALAT---TRHRLARLQREGDDGQG-FAQLI
Sbac_MBN51294.1       LTH---APEEEQDCFSDDTHNSLWYNAKGIENFYYARYEGYSSFHA-PERSLSALAKRY-YPELAGELDQAFTQ---TRVAMQAIREQGESHQQFVDQLI
Ssp._WP_011619424.1   ASG----KGELESTFSGNTGADVLSNLQGVQLAWEKS-------------KIQEYAKTK-DAELSNQLTVELQT---ALAQAKELPTR-------LNDKL
CSsp_KKZ15492.1       EDG----PEALESRFSGTTNADIVANVSGAQRVFEDT-------------GLKQLLAAK-DADLAKQLSDSLAM---SVSATEAMPSR-------LSQRL
Lkob_WP_109018696.1   ASG---SQEEEHSCFSDNTKNDFYYDATGFENIFTGTYNGS--SVS-PGIGLGSILTSE-EKTTTLKNISNAKL(7)EDPSLNQTCANGTISYR-FDQVI
Bbac_PCH67655.1       DTK---NVLEVESWYSFNSLIDFTNNIYSIENAYLGGYDAE-----TRGENLSGAVKAK-NEALDQKVRNQITA---TIAAINAIKYP-------FRDQL
Hmar_CBW26097.1       LLE---DQEEEHSCFSDQTINDIYTNALGVKNIYFGDYVAHNSSKSINGKGINDLVAFV-NPELDQAITKKFDS---LFNNINFFYKKSRNGQV-QIGSI
Consensus/80%         ......s.cpEpssFSDsTb.sbb.shbGbbs.ahGpY..........s.sb.pbh....s..hs.pbp..b.....s...h.hb.........ac.bb

Secondary Structure            HHHHHHHHHHHHHHHHHHHHHHHHHHHHHH
CLas_WP_102134465.1   SENN---PEGNKIVRNLINDLITQTESLRKIRIALDLIEP
CLaf_WP_052775026.1   SDNN---PEGNKIVRNIIDDLITQTESLKKLRIALDLKQT
CLso_WP_044054258.1   SANN---PEGNKIVLNLIDDLITQTNSLKKLRIALDLKEI
CLam_WP_007556626.1   NDNN---PIGNKIVRNVIDDLIRQTESLRKLRIVFDLKEA
CLeu_PTL86891.1       SSNN---PIGNKIVQNVINDLVEQTESIKKLRIVLDIEDT
Rcel_WP_148146048.1   GENN---AKGNAVVQKAIDGLIDQTKSIERVIASLDLGKI
Avit_WP_087727067.1   AENN---KEGNATVQAGIDGLIDQTKSIQRVVAALDLGEV
Vbac_MAS96703.1       QSGN---EEGRAVLNELISRLKAQTELIEKVHMMVPELAK
Chut_ABG57953.1       LTAN---TAGTIRVQNAINALHVEAAQYKNAANKIGYLY-
Pbac_PZN34535.1       QPDN---EAGRARVRAAIDALRAQSLSMFRAAEAMQIFLS
Mbac_RYE76643.1       LEAD--GSPNRARAIEAITALKSASDGLVQGANKLGLAI-
Asp._PHR58916.1       IPGA---------DADRIKALATELVTMGNMMVNVGTDIG
Tcin_GEY51124.1       AAGP(7)LPRRQNLQQAVFVLPLRWPRPGATSRGRVPRSV
Psp._WP_158799209.1   DGSN---AAGNLIVQKGVTDVSAVADWVSQIAHDLNLTNV
Gbac_PIE39618.1       --------QTTANVYKTIIALNQQSASIAALAEKLAIEVK
Plut_KZN30969.1       QTE-----KHQLEVRAAMTALKDQTAIVQKVIDTLGLETE
Tasi_WP_015551911.1   LPQN---EVHNKNLVSMKNQMRRLGDQMIAVAKSLHIDLT
Llut_WP_146646404.1   VGDD--ASPGRKAIAAAVDSLRAQGDLFAEAASVLGLSIK
Nbac_MBV62577.1       APEN---VQGSRLLADAMIALRVQSALLSRLGEALGLGPL
Sbac_MBN51294.1       APDN---AAGNALLQSAIDRLQAQARLLNRLARNLGIELM
Ssp._WP_011619424.1   DN-----GDTQKQVKMLMEAISKAFDTTEALKSKIG----
CSsp_KKZ15492.1       DD-----PAAVESMEMAIEATESAFNLMTQALETLDSF--
Lkob_WP_109018696.1   VASAV-GTTQHTIFTGTIQTSVKAIGKTIQAAAKRFGTTI
Bbac_PCH67655.1       DN-----TGEYADIEAAIKACDDLRAILEDEVKALFVY--
Hmar_CBW26097.1       ETPF(4)TTNKVEIQNIIDDLGSIDELLREAASELGLEIE
Consensus/80%         .s........p..h..hbpsb..b...b.ph...bsh...
```

HHH → **alpha helix**

EEE → **beta strand**

Supplementary Figure 9. Multiple sequence alignment (MSA) of the Phage_lysozyme (PF00959) family. For coloring themes refer to the Supplementary Figure 3 legend.

```
Secondary Structure       HHHHHHHHHHH      EEEEEE        EEEE                    HHHHHHHHHHHHHHHHHHHHHH            HHHHHHHHHH
CLas_WP_015452887.1   IPVPNALIKMLKEFEG---LRLTAYRDIGG--GAWTIGYGHTGS----DVTEGMTITEKEAEDFLLKDASKSLNLLLESSPAL---KSTSENRLVAVADF
CLaf_WP_047264530.1   VLIPGLLVDMVERYEG---VRVTAYKDPGR--GVWTIGYGHTGP----DVHEGMTITQEQAEELLKGDLRKHVDLLLSASPIM---ASVSENRLAAVGDF
CLso_WP_013461579.1   --MPHLLIDLVKGFEG---LRLKAYRCSA---GIWTIGYGHTGN----DVFENLAITEKQANDLLKWDVSKCLSQVFTVSPIL---INAGENRISAIGDF
CLso_WP_013462155.1   IIIPPLLLNLIKRFEG---QRLKAYQCPA---GVWTIGYGHTGN----DVFKDLVITEQKAESLLKQDVLKFLTQVFKISPSL---IDAGENRISAIGDF
CLso_WP_044054126.1   ----------MKRFAG---LRLTAYRCSA---GIWTIGYGHTGN----DVFENLAITEQQADDLLKWDVSKCLSQVFTVSPIL---IHAGENRISAIGDF
CLam_WP_007557487.1   ITIPQLLISLIKQFEG---LRLKAYRCSA---GVWTIGYGHTGC----DVYDGLVITQEQAENLLNYDIRRHLNVTIKLSPSL---LSSNDNCLSAIGDF
CLeu_PTL86076.1       -----MLLDLIIEFEG---LRLKSYQDPA---GVWTIGYGHTGP----DVVEGMVITQPQVYEFLIQDASQCIRQTFEVSPII---KECGANRISAIGDF
CLeu_PTL86791.1       TTIPSLLIDVIKNFEG---LRLKAYRCPA---GVLTIGYGHTGF----DVCEDMNISEQHADELLILDVSKCLNQVLNVSPIL---INANENRISAIGDF
Abau_6ET6_A           KTTSDDGIDLITSFEG---TRFNAYDDGV---GVWTIGTGTTVYPNGVKVKKGDTCTAEQAKTYFKHDLAKFEKTVNESVT-----APLTQNQFDALVSL
Khan_WP_048859119.1   MNLPTYLIQLVESSEG---LRLEPYQDCC---GVWTQGYGTTGSR----IKPGQAITPEQAQEWLISDLEAIWQAITARSTIQ-----LETYQTAALADF
Atro_WP_039904868.1   MDIPETLVELVAEFEG---FKAEAYQDIG---GVWTCGYGTTGPD----IHKGTTFTKEEALQRLSETLQSIWNQIEQKTSIA-----LTSKQMEALSSF
Dpig_WP_204673942.1   AFDGPDLLTITKHYEG---FKAHPYLCPA---GVWTIGYGHTEGV----SRNNPPVNKEEAEQLLIKDLLVATHAVLRHAGPA(6)PEEAYRRLVALASW
Dsp._WP_009303990.1   DIFNHPMTEAVKHYEG---LRLVPYKCPA---GKWTIGYGHLCP------PDQRPISKEEAERLLRWDMLDAFGAMLRQWPEG---ADAPAHQLAALTSF
Pshi_WP_010864476.1   LKVSDEGLKLIADFEG---CRLTPYRCSA---GVWTNGIGHTEG-----VTSKSVVTERQVAENLVADVAR-TEKALAH-CMP---VTMPQPVYDAVVSW
Pzuc_PZQ57970.1       -AIIAAAVKLAKPFEG---WRARPYLCPA---RVWTQGYGSTKG----VKPTNPPWSREHGEAVISEEMDT-FAGALLAYSPN--LKAQHPDVGGALLDF
uCau_CAB4164981.1     -MIPDLAIALIARFEG---FSAKPYLCPA---GKWTIGYGTLCR------PDHPPVTEEEAQALLIGYLER-VAPHILVQAPA---LENSEARLAAVLSW
Maer_NCR22735.1       --IPQRAIELIKEFEG(7)GRVQAYPDPN(5)KLPTIGYGSTRCP(4)-VRKGQIITRDEAEEYLMYDLDKIRRPALEKIPTW---KDMNENQRAALYSF
Lsp._NEQ54273.1       --VPQAAIDLIKRFEG(9)DRVKAYPDPG(4)SLPTIGYGTTYYP(4)-VQQNDIITREESERFLIHEVEEKCRLALETIPTW---GQMNPNQRAALYSF
uCau_CAB4240787.1     ---MKDLLILIKRFEG---CRLKAYFCPA---GILTIGWGSTGG-----ISIGDIWTQEQADKRLEQDTYK-FFVGVSKLIPN-----ASDNVKIACSDF
Bbac_MBE6304922.1     ---MENAISLIKRFEG---LSLSAYRCPA---GVWTIGWGHTKN-----VRPLMSVSHAEADELLRNDVSE-IFTKLTQLSAE-ANVTLSSNQLSALTSF
Mbac_GFI06907.1       -----MIYNLIRRHEG---LRLHAYRCEG---GMWTVGYGHTAD-----VNAGTTVTEDEAMRLLREDVAE-VRGRIEHMCAQ-DGVTLSAVQADALTSF
Bbac_MBD5239742.1     ------MKDLIKRFEG---LRLEAYRCAA---GVWTIGWGHTAG-----VTAGMKITEAEAERLLDEDLRP----VVAALPPG-----LSDNQRAALASL
Bbac_MBE6293835.1     -KISDYAFEKIKDFEG---LRLKAYLCSG---GKWTIGYGHTKG-----VHEGMVITRQEADRLLEEDILY-FENFLSKEKYA---EDITQGQWDALVSF
Bbac_NLA94747.1       MKATNYATLLIKSFEA---LRLNSYLCPA---GRWTIGYGHTEG-----VNQGMLITEKTADAFLRQDIHH-AEAEVNRIDAD-----LTQEQFDALVSF
Bbac_OJV19870.1       MKVSQQGVQLIKESEG---LRLQAYRCPA---GKWTIGYGHTKK-----ARAGMVITADKAEQLLQEDIRD-IEGTLNNSGLI-----LTQNQFDAFADF
uCau_CAB4127944.1     MEISKNGLDLIKTFEG---CKLKAYLCPA---KVWTIGYGATYYE(4)-VKAGDVITEERAVGLLKKLVAN-FYHEIKG---------INQNQFDAITSF
Bsp._WP_002562728.1   MKTSESAKQAIGTFEG---LELKAYLCPA---GVWTIGYGHTKG-----VHKGMQITKEQALAFLSSDLAD-VERNLNTRFPS-----ISQNKFDAMISL
Bbac_MBE6322682.1     MRASDNAKALIKSYEG---LRLKAYKCPA---GVWTIGYGHTKG-----VTQGMEISRQMAEGYFEQDLYNIAEYPISDIFYK-AKVTLTQNQFDALCSF
Consensus/80%         ......hhpbhppaEG...hchpsYbsss...GhWTIGYGpT.......h..s..hspppA..bL..Dh.p.h...bp..s.......hs.spbsAbssa

Secondary Structure   HHHH HHHHH     HHHHHH    H   HH HHHH H     EE      E   E HHHHHHHHHHHHHH
CLas_WP_015452887.1   VFNLGIGNYN---KSTFKQRV---DAQDWE-KAAE-ECKKWTKA---GGK---VLPGLVKRRDAEVKLLLE--
CLaf_WP_047264530.1   VFNLGIGNYR---KSTFKKCI---DAEDWE-NAAT-ECKKWIHG---GGK---VLPGLVKRRNEEAALLISG-
CLso_WP_013461579.1   VFNLGIGRYR---NSTLRKRV---DREDWI-NASH-EICKWVFA---GGK---KLKGLVIRREIEADLLLKSE
CLso_WP_013462155.1   VFNLGIARYR---KSTLRKRV---DVGDWK-SASD-ECKKWCFA---GQK---KLRGLVLRRKVEADLLLKG-
CLso_WP_044054126.1   VFNFGIGRYR---ASALRKCV---DAEDWV-TASH-EIRKWVFA---GGK---KLNGLVLRGEVEAELLLKN-
CLam_WP_007557487.1   VFNLGTTRYK---NSTLRRCV---NSGQWE-EASQ-ECRRWVYG---GGK---RLKGLVARRAIEAELLLGG-
CLeu_PTL86076.1       VYNLGIGNYR---SSTLRKRV---DVKDWD-GAVD-EIKKWVHV---GKK---TLPGLVRRRNSEACLLLMG-
CLeu_PTL86791.1       VFNLGIGRYK---ASTFRKRV---DEEDWD-EASK-ECTRWVFA---GGR---KLKGLVARREIEAALLLQC-
Abau_6ET6_A           TYNIGSGAFN---NSTLLKKL---NKGDYQ-GAAD-QFLVWNKA---GGK---VMKGLVRRREAERALFLK--
Khan_WP_048859119.1   AYNLGINALF---KSTLWQLV---QANQPI-NASL-EFSKWVYA---GKH---IEPGLVIRRHKEALIFLG--
Atro_WP_039904868.1   AYNLGTSALF---KSTLWKYL---EEGEIE-QAAQ-EFPKWSHV---NGK---VITGLLKRRVKEQELFLS--
Dpig_WP_204673942.1   TFNLGEGNLS---SSTMLKRI---RVLDWD-AAAN-EMLRWDKA-TVGGR-TVALPGLTKRRKTEAHYFRT--
Dsp._WP_009303990.1   VFNLGEGNFA---SSTLLQRL---RARKFS-DVPA-QIRRWNKATVDGVK--KRLPGLVKRRQCEALCFEAGE
Pshi_WP_010864476.1   GFNVGPSAAC---RSTLAHFI---NQRNWS-QACQ-QLSRWVYV---GGV---KNAGLVSRRQRELAHCLR--
Pzuc_PZQ57970.1       VFNLGPTAYK---ASTLRRKV---AAEDWE-EVEY-QLQRWVFG---GGR---KLPGLVRRRKAEGDQIGA--
uCau_CAB4164981.1     VYNLGLGNFT---ASTFRRCL---AAQDWP-GAAF-QCRRWSRA---GGK---RLAGLVARREVEARLLCG--
Maer_NCR22735.1       SFNLGSGFYR(9)VCDSPQRW---NDQEWV---KE-QFVKYRNP---GTN---VESGLRRRREAEAKLFCT--
Lsp._NEQ54273.1       AYNLGAGFYG(9)VCDSPRKW---NDKQWV---AE-QFGKYVRS---NNE---VLPGLVRRRAAEAQLFCK--
uCau_CAB4240787.1     AYNLGLGAFK---TSTLRRKI---LAGDIE-GAKM-QLARWNKS---GGK---ILKGLTLRRQAEIAYI----
Bbac_MBE6304922.1     IFNVGFSAFR---RSTLWKLI---RENPDNPDIAR-QFAKWKYA---AGK---VMSGLVRRRREEATLYFK--
Mbac_GFI06907.1       AFNVGTEALR---RSTLWSLV---KRDPGDPAIAR-EFSRWVYG---GGR---RLPGLVKRREEEAALYFS--
Bbac_MBD5239742.1     CFNIGISAFL---RSTIRRKV---CANPADPSIAA-EFLRWKYS---RGR---ELPGLLKRRRAEAEVYMS--
Bbac_MBE6293835.1     IFNLGIGNFI---CSTLRNKIL--INIDEP-TIPD-EFRKWVFA---KGK---KLQGLVKRREWEAQMYEW--
Bbac_NLA94747.1       VFNVGIQAFR---VSTLRKLV---ERNPSDPKIAD-EFRRWVYA---GDK---KLPGLIKRREQEIKLYYS--
Bbac_OJV19870.1       IFNFGAPKFL---SSTFYRYAKA-SSSDPR--IPA-ELRRWVHS---TDD(5)VQPGLVKRREREIALYLK--
uCau_CAB4127944.1     CYNLGNGNFN---ASTLKKKI---VANPNDPSIEA-EFLKWNKA---GGK---VLKGLTIRRSVEWKLYSK--
Bsp._WP_002562728.1   SFNIGIQAFN---TSTLYRKAKA-NLNDPS--IRI-EFMKWVHS---KGK---VLPGLVERRTWEANLYFS--
Bbac_MBE6322682.1     VYNCGMGNFK---KSTLLKKAL-ANPND---KSIYCEFMKWNKA---GGK---EVTGLTNRRKKEAELYFK--
Consensus/80%         saNbG.s.a.....STbbpbh...p..p....h...ph.cWsbs...ssc....b.GLsbRRp.Eh.bbh...
```

HHH → alpha helix

EEE → beta strand

Supplementary Figure 10. Multiple sequence alignment (MSA) of a new protein family which contains four conserved Cysteines. For coloring themes refer to the Supplementary Figure 3 legend.

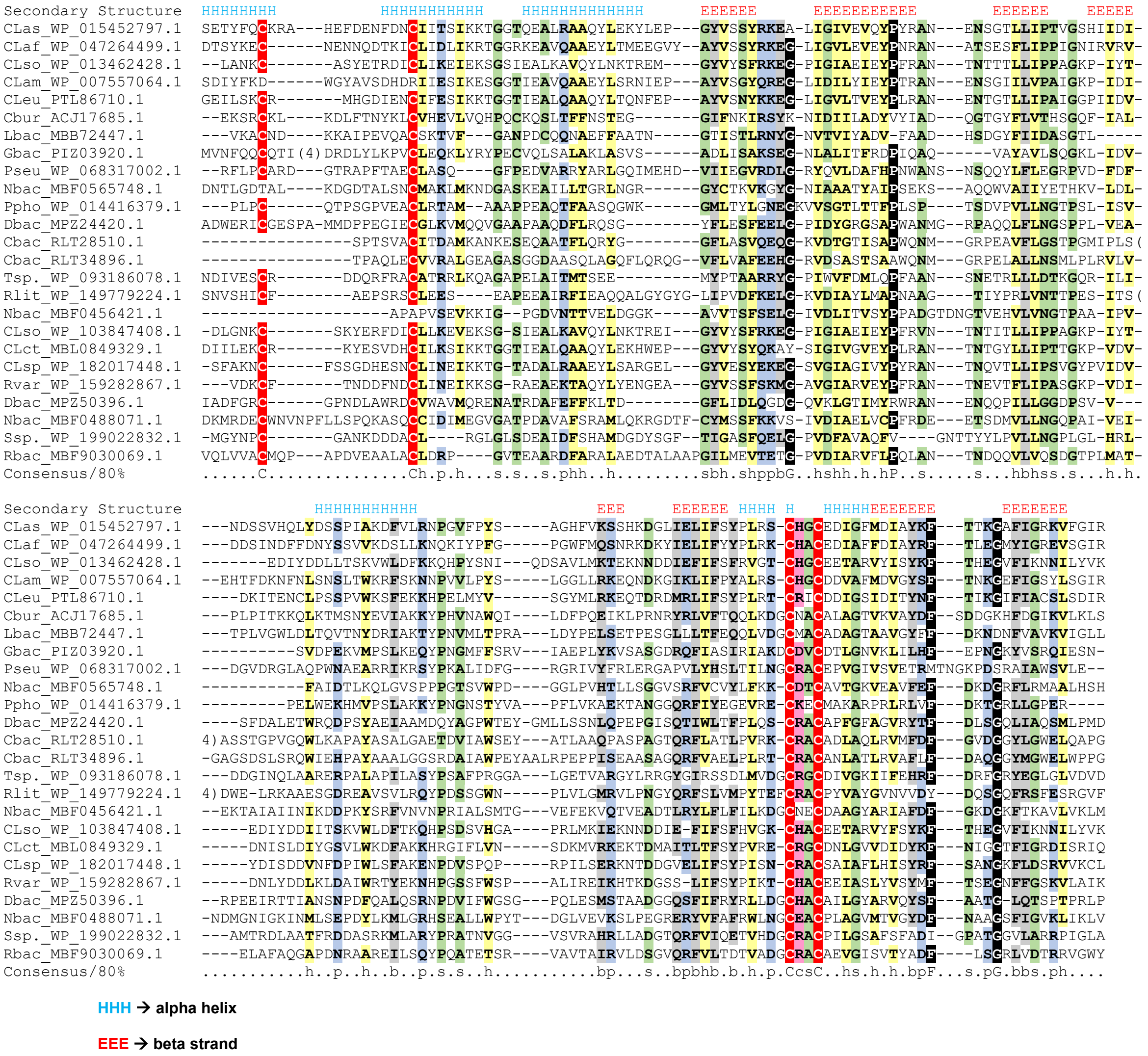

Reference

1 Lin, H. *et al.* Complete genome sequence of a Chinese strain of “Candidatus Liberibacter asiaticus”. *Genome announcements* **1** (2013).

2 Duan, Y. *et al.* Complete genome sequence of citrus huanglongbing bacterium,‘Candidatus Liberibacter asiaticus’ obtained through metagenomics. *Molecular Plant-Microbe Interactions* **22**, 1011-1020 (2009).

3 Zheng, Z., Deng, X. & Chen, J. Whole-genome sequence of “Candidatus Liberibacter asiaticus” from Guangdong, China. *Genome Announcements* **2** (2014).

4 Zheng, Z. *et al.* A type 3 prophage of ‘Candidatus Liberibacter asiaticus’ carrying a restriction-modification system. *Phytopathology* **108**, 454-461 (2018).

5 Katoh, H., Miyata, S.-i., Inoue, H. & Iwanami, T. Unique features of a Japanese ‘Candidatus Liberibacter asiaticus’ strain revealed by whole genome sequencing. *PLoS One* **9**, e106109 (2014).

6 Dai, Z. *et al.* Prophage diversity of ‘Candidatus Liberibacter asiaticus’ strains in California. *Phytopathology* **109**, 551-559 (2019).

7 Lin, H. *et al.* Complete genome sequence of “Candidatus Liberibacter africanus,” a bacterium associated with citrus huanglongbing. *Genome announcements* **3** (2015).

8 Lin, H. *et al.* The complete genome sequence of ‘Candidatus Liberibacter solanacearum’, the bacterium associated with potato zebra chip disease. *PLoS One* **6**, e19135 (2011).

9 Wulff, N. A. *et al.* The complete genome sequence of ‘Candidatus Liberibacter americanus’, associated with citrus huanglongbing. *Molecular Plant-Microbe Interactions* **27**, 163-176 (2014).

10 Frampton, R. A. *et al.* Draft genome sequence of a “Candidatus Liberibacter europaeus” strain assembled from broom psyllids (Arytainilla spartiophila) from New Zealand. *Genome announcements* **6** (2018).

11 Leonard, M. T., Fagen, J. R., Davis-Richardson, A. G., Davis, M. J. & Triplett, E. W. Complete genome sequence of Liberibacter crescens BT-1. *Standards in genomic sciences* **7**, 271-283 (2012).

12 Mol, C. D., Kuo, C.-F., Thayer, M. M., Cunningham, R. P. & Tainer, J. A. Structure and function of the multifunctional DNA-repair enzyme exonuclease III. *Nature* **374**, 381-386 (1995).

13 Romier, C., Dominguez, R., Lahm, A., Dahl, O. & Suck, D. Recognition of single-stranded DNA by nuclease P1: High resolution crystal structures of complexes with substrate analogs. *Proteins: Structure, Function, and Bioinformatics* **32**, 414-424 (1998).

14 Buggy, J. & Bauer, C. E. Cloning and characterization of senC, a gene involved in both aerobic respiration and photosynthesis gene expression in Rhodobacter capsulatus. *Journal of bacteriology* **177**, 6958-6965 (1995).

15 Xu, Q. *et al.* Structural and sequence analysis of imelysin-like proteins implicated in bacterial iron uptake. *PLoS One* **6**, e21875 (2011).

16 Foster, P. G., Nunes, C. R., Greene, P., Moustakas, D. & Stroud, R. M. The first structure of an RNA m5C methyltransferase, Fmu, provides insight into catalytic mechanism and specific binding of RNA substrate. *Structure* **11**, 1609-1620 (2003).

17 Busam, R. D. Structure of Escherichia coli exonuclease I in complex with thymidine 5′-monophosphate. *Acta Crystallographica Section D: Biological Crystallography* **64**, 206-210 (2008).

18 Scheerer, P. *et al.* The class III cyclobutane pyrimidine dimer photolyase structure reveals a new antenna chromophore binding site and alternative photoreduction pathways. *Journal of Biological Chemistry* **290**, 11504-11514 (2015).

19 Morita, M. *et al.* Antibacterial activity of Bacillus amyloliquefaciens phage endolysin without holin conjugation. *Journal of bioscience and bioengineering* **91**, 469-473 (2001).
20 Antonyuk, S. V. *et al.* Structure of SurE protein from Aquifex aeolicus VF5 at 1.5 Å resolution. *Acta Crystallographica Section F: Structural Biology and Crystallization Communications* **65**, 1204-1208 (2009).
21 Carfi, A. *et al.* The 3-D structure of a zinc metallo-beta-lactamase from Bacillus cereus reveals a new type of protein fold. *The EMBO journal* **14**, 4914-4921 (1995).
22 Leesong, M., Henderson, B. S., Gillig, J. R., Schwab, J. M. & Smith, J. L. Structure of a dehydratase–isomerase from the bacterial pathway for biosynthesis of unsaturated fatty acids: two catalytic activities in one active site. *Structure* **4**, 253-264 (1996).
